# Supplementary material for: Optimal three-part tariff pricing and marketing strategies for consumer overconfidence
Source: PLoS One. 2024 Nov 20;19(11):e0297819. doi: 10.1371/journal.pone.0297819 (PMC11578462; doi:10.1371/journal.pone.0297819)
Supplement: S1 Appendix — (PDF) [file pone.0297819.s001.pdf]

Appendix 1: Profit maximization model

A. Profit maximization when the fixed fee (T) is less than $\mathrm{CS}(\mathrm{O_1})$

The service is purchased by all the consumers within the interval $(\mathrm{O}^*, 1)$. The expected consumption Q is represented as follows:

$$\mathrm{Q} = \int_{\mathrm{O}^*}^{\mathrm{O}_1} \mathrm{O} * \mathrm{f(O)\,dO} + \mathrm{q_0} * \int_{\mathrm{O}_1}^{\mathrm{O}_2} \mathrm{f(O)\,dO} + \int_{\mathrm{O}_2}^{1} \left(\mathrm{O} - \frac{\mathrm{p_0}}{\lambda}\right) \mathrm{f(O)\,dO}$$

$$= \frac{1}{2} + \frac{\theta_1^2}{2} - \frac{p_0(1-\theta_2)}{\lambda} - \frac{\theta_2^2}{2} + q_0(-\theta_1 + \theta_2) - \frac{(\theta^*)^2}{2}$$

$$= \frac{1}{2} - \frac{(\sqrt{2}\sqrt{T} - r\sqrt{\lambda})^2}{2\lambda} - \frac{p_0(1 - r - \frac{p_0}{\lambda} - q_0)}{\lambda} + \frac{p_0 q_0}{\lambda} + \frac{1}{2}(r + q_0)^2 - \frac{1}{2}(r + \frac{p_0}{\lambda} + q_0)^2. \quad \text{(A1)}$$

Only the consumers within the range $(\mathrm{O}_2\,, 1)$ consume more than the allowance. Thus, Qe is represented as

$$\mathrm{Qe} = \int_{\mathrm{O}_2}^{1} (\mathrm{O} - \frac{\mathrm{p_0}}{\lambda} - \mathrm{q_0})\,\mathrm{dO}$$

$$= \frac{1}{2} + (-\frac{p_0}{\lambda} - q_0)(1 - \theta_2) - \frac{\theta_2^2}{2}$$

$$= \frac{1}{2} + (-\frac{p_0}{\lambda} - q_0)(1 - r - \frac{p_0}{\lambda} - q_0) - \frac{1}{2}(r + \frac{p_0}{\lambda} + q_0)^2. \quad \text{(A2)}$$

To maximize profits, the monopolistic service provider will adjust T, $\mathrm{p_0}$, and $\mathrm{q_0}$. Profits can be classified into two types, that is, from the fixed fee and from the usage price, as shown in Equation (8).

$$\mathrm{Profit} = \mathrm{T} * \mathrm{N} + \mathrm{p_0} * \mathrm{Q_e} - \mathrm{mc} * \mathrm{Q}$$

$$= p_0(\frac{1}{2} + (-\frac{p_0}{\lambda} - q_0)(1 - \theta_2) - \frac{\theta_2^2}{2}) + T(1 - \theta^*) - \mathrm{mc}(\frac{1}{2} + \frac{\theta_1^2}{2} - \frac{p_0(1-\theta_2)}{\lambda} - \frac{\theta_2^2}{2} + q_0(-\theta_1 + \theta_2) - \frac{(\theta^*)^2}{2})$$

$$= T(1 - \frac{\sqrt{2}\sqrt{T} - r\sqrt{\lambda}}{\sqrt{\lambda}}) + p_0(\frac{1}{2} + (-\frac{p_0}{\lambda} - q_0)(1 - r - \frac{p_0}{\lambda} - q_0) - \frac{1}{2}(r + \frac{p_0}{\lambda} + q_0)^2) - \mathrm{mc}(\frac{1}{2} - \frac{(\sqrt{2}\sqrt{T} - r\sqrt{\lambda})^2}{2\lambda} - \frac{p_0(1 - r - \frac{p_0}{\lambda} - q_0)}{\lambda} + \frac{p_0 q_0}{\lambda} + \frac{1}{2}(r + q_0)^2 - \frac{1}{2}(r + \frac{p_0}{\lambda} + q_0)^2)$$

$$= \frac{1}{2\lambda^2}(\lambda(-2\sqrt{2}T^{3/2}\sqrt{\lambda} + 2(1 + r)T\lambda + \mathrm{mc}(2T - 2\sqrt{2}r\sqrt{T}\sqrt{\lambda} + (-1 + r^2)\lambda)) + p_0^3 - p_0^2(\mathrm{mc} + 2\lambda - 2\lambda q_0) + \lambda p_0(2\mathrm{mc} + \lambda - r^2\lambda - 2(\mathrm{mc} + \lambda)q_0 + \lambda q_0^2)). \quad \text{(A3)}$$

Then, we differentiate the profit function with respect to T, p_0, and q_0 to determine the optimal value of each endogenous variable. Below, the equations represent first-derivative conditions.

1. $\frac{\partial \Pi}{\partial T} = 0 \rightarrow 1 + r + \frac{mc}{\lambda} - \frac{mcr}{\sqrt{2}\sqrt{T}\sqrt{\lambda}} - \frac{3\sqrt{T}}{\sqrt{2}\sqrt{\lambda}},$ (A4)
2. $\frac{\partial \Pi}{\partial p_0} = 0 \rightarrow \frac{1}{2\lambda^2}(3p_0^2 - 2p_0(mc + 2\lambda - 2\lambda q_0) + \lambda(2mc + \lambda - r^2\lambda - 2(mc + \lambda)q_0 + \lambda q_0^2)),$ (A5)
3. $\frac{\partial \Pi}{\partial q_0} = 0 \rightarrow \frac{2\lambda p_0^2 + \lambda p_0(-2(mc+\lambda)+2\lambda q_0)}{2\lambda^2}.$ (A6)

We substitute Equation (A5) into (A6) and solve Equation (A4) to obtain the best solution for q_0, p_0, and T. Thus, the optimal solution of each endogenous variable can be specified as

$$q_0 = 1 + \frac{mc}{\lambda} - \frac{\sqrt{mc^2 + r^2\lambda^2}}{\lambda}, \tag{A7}$$

$$p_0 = \frac{1}{3}\left(-mc + 2\sqrt{mc^2 + r^2\lambda^2} + \sqrt{5mc^2 + 4r^2\lambda^2 - 4mc\sqrt{mc^2 + r^2\lambda^2}}\right), \tag{A8}$$

$$T = \frac{1}{18}\Bigg(4mc - 2mcr + \frac{2mc^2}{\lambda} + 2\lambda + 4r\lambda + 2r^2\lambda + \sqrt{-36mc^2r^2 + \left(-4mc + 2mcr - \frac{2mc^2}{\lambda} - 2\lambda - 4r\lambda - 2r^2\lambda\right)^2}\Bigg). \tag{A9}$$

We obtain the maximum expected profit by including the above optimal values of the endogenous variables into Equation (8).

B. Profit maximization when $CS(O_1) < T < CS(O_2)$

The service is purchased by all the consumers within the interval $(O^*, 1)$. The expected consumption Q is represented as

$$Q = q_0 * \int_{O^*}^{O_2} f(O)\, dO + \int_{O_2}^{1} \left(O - \frac{p_0}{\lambda}\right) f(O)\, dO$$

$$= \frac{1}{2} - \frac{p_0(1-\theta_2)}{\lambda} - \frac{\theta_2^2}{2} + q_0(\theta_2 - \theta^*)$$

$$= \frac{1}{2} - \frac{p_0(1 - r - \frac{p_0}{\lambda} - q_0)}{\lambda} - \frac{1}{2}(r + \frac{p_0}{\lambda} + q_0)^2 + q_0(r + \frac{p_0}{\lambda} + q_0 - \frac{2T - 2r\lambda q_0 - \lambda q_0^2}{2\lambda q_0}). \tag{B1}$$

Only the consumers within the range $(O_2, 1)$ consume more than the allowance; thus, Qe is represented as

$$\mathrm{Qe} = \int_{\mathrm{O}_2}^{1} \left(\mathrm{O} - \frac{\mathrm{p}_0}{\lambda} - \mathrm{q}_0\right) \mathrm{dO}$$

$$= \frac{1}{2} + (-\frac{p_0}{\lambda} - q_0)(1 - \theta_2) - \frac{\theta_2^2}{2}$$

$$= \frac{1}{2} + (-\frac{p_0}{\lambda} - q_0)(1 - r - \frac{p_0}{\lambda} - q_0) - \frac{1}{2}(r + \frac{p_0}{\lambda} + q_0)^2. \quad \text{(B2)}$$

To maximize the expected profit, the monopolistic service provider can adjust T, p_0, and q_0. As stated above, the two types of profits are from the fixed fee and from the usage price, as shown in Equation (B3).

$$\mathrm{Profit} = \mathrm{T} * \mathrm{N} + \mathrm{p}_0 * \mathrm{Q}_\mathrm{e} - \mathrm{mc} * \mathrm{Q}$$

$$= p_0(\frac{1}{2} + (-\frac{p_0}{\lambda} - q_0)(1 - \theta_2) - \frac{\theta_2^2}{2}) - \mathrm{mc}(\frac{1}{2} - \frac{p_0(1 - \theta_2)}{\lambda} - \frac{\theta_2^2}{2} + q_0(\theta_2 - \theta^*)) + T(1 - \theta^*)$$

$$= p_0(\frac{1}{2} + (-\frac{p_0}{\lambda} - q_0)(1 - r - \frac{p_0}{\lambda} - q_0) - \frac{1}{2}(r + \frac{p_0}{\lambda} + q_0)^2) + T(1 - \frac{2T - 2r\lambda q_0 - \lambda q_0^2}{2\lambda q_0}) - \mathrm{mc}(\frac{1}{2} - \frac{p_0(1 - r - \frac{p_0}{\lambda} - q_0)}{\lambda} - \frac{1}{2}(r + \frac{p_0}{\lambda} + q_0)^2 + q_0(r + \frac{p_0}{\lambda} + q_0 - \frac{2T - 2r\lambda q_0 - \lambda q_0^2}{2\lambda q_0})). \quad \text{(B3)}$$
