# Supplementary material for: Optimal three-part tariff pricing and marketing strategies for consumer overconfidence
Source: PLoS One. 2024 Nov 20;19(11):e0297819. doi: 10.1371/journal.pone.0297819 (PMC11578462; doi:10.1371/journal.pone.0297819)
Supplement: Attachment — Submitted filename: Review of the paper optimal tariff pricing and marketing strategies for consumer overconfidence.docx [file pone.0297819.s003.docx]

Review of the paper optimal tariff pricing and marketing strategies for consumer overconfidence

Summary

This paper attempts to understand how firms may determine the optimal three-part tariff structure when facing overconfident consumers. Overconfident consumers tend to miscalculate their future consumption which may be used by firms to maximize profits. Firms may charge a higher fix cost for a specific number of unit allowance and a small usage fee for units consumed above that specified number, expecting overconfident consumers to pay the fix cost, and not consuming all the units’ allowance. In this context, the paper looks for the optimal parameters and the optimal menu of three parts tariff maximizing firms’ profits.

Main comments

- My first comment must be about how the paper has been written. The paper is very confusing to read, some parameters of the model are not well explained (for instance, the role of \theta_1 and \theta_2), and there seems to be a changed of variable at some point about the consumer types (being \theta at the beginning of section 2 and then becoming \theta+r in my interpretation). Many other typos of this type are found along the paper, but I will not mention all of them. I think is the role the authors to provide a paper more carefully written making the role of the reviewers easier.
- Other important issue to mention is that the paper does not really focus on the optimal three-part tariff structure because their solution depends on several specific parameters and assumptions. In the best-case scenario, they can find some local optimums but not the global optimum of the problem. This is even the case with the algorithm approach. This concern it's more problematic given the nonlinear nature of the profit functions they have.
- These nonlinearities are also relevant for comparative static analysis where the authors use only two different values for the marginal cost and the consumer price sensitivity. The effect found cannot be generalize to a different range of numbers and the authors do not explain why the numbers selected are the most relevant in this context. In particular, the price sensitivity equal 1.
- I am aware that the difficulties to solve these types of models open the door to this type analytical and numerical approaches, but they are usually a tool used to understand the role of a particular mechanism. However, the mechanism in your paper was already known and your contribution is to find the optimum three-part tariff. In my opinion, this methodology does not allow you to solve this problem.
- Respect to the assumptions of the model, I wonder why the authors assume that the level of overconfidence is the same for all consumers in the population. Moreover, they assume that there is no relationship between their types and their level of overconfidence. Of course, these assumptions help to maintain the uniform distribution of types and the tractability of the model, but it also limits its applications. Could the authors extend their analysis to other types of distributions?
- At the beginning, the model assumes that consumer types are uniformly distributed on the range between zero and one. However, in the profit maximization of the firm the consumers are distributed between \theta star and \theta upper bar. Similarly, in the numerical analysis consumer types are not between zero and one. The uniform distribution between zero and one make it easier to calculate the consumer surplus but then you cannot change the range of the distribution. the authors should explain better the reasons behind this change.

Minor comments

- Respect to the analysis of the number three-part tariffs in the menu offered by the firm, it is not surprising that you find dot a menu with more options dominates the others. Basically, more pricing options allow you for a better discrimination of the consumers and therefore higher profit. Considering this a more interesting analysis will be to determine when this discrimination capacity matters the most.
- The authors use the demand function coming from Tirole (1995) and they argue that they're reasonable assumptions for the appropriateness of this demand function in terms of utility theory. I think the authors should discuss those assumptions in the paper and to discuss how appropriate is this function to study overconfidence.
- More explanation is needed in the genetic algorithm. What determines the selection of individuals in each iteration? Do you use any measure of fitness in this case, or everything is random from the original population distribution?
- It is important to differentiate throughout the paper between the consumer surplus and expected consumer surplus, based on the difference between consumption and expected consumption that the authors do not make.
- In section 2.1, you include a double characterization of the consumers. First, you mentioned that there are two types of individuals, \theta_1 and \theta_2, but then you use these two types to characterize regions of consumers. The explanation is confusing, and you should differentiate the role of these categories better.
- The last paragraph section 2.2 we shall have \theta+r in many places, it looks like a mistake. But an alternative explanation is that they are using \theta as \theta+r which is confusing giving his presentation of the model in Section 2.
- In the parametrization of the model, which are reasonable levels of overconfidence a why?
- Table 2 and figure 2 show the same information they should move one of them to the appendix.
- A sensibility analysis is needed in the simulation respect to the main parameters to determine in what range your conclusions are valid.
- In section 2.3, the firm should not maximize respect to marginal costs that's something that you should take as given.

To conclude, I think the paper fails to find the optimal three-part tariff structure with overconfident consumers given my comments above. For this reason, I suggest rejecting the paper.

Section 2

moreover they assume that there is no relationship with consumer type and their own level of confidence

they also mentioned that the type of consumers go from zero to 1 but then in the air graphical analysis they have but it'll lower bar and a theater upper bar upper bar

In section 2.3 I do not understand why the density function is integrated between Theta star and T to upper bar if the distribution of consumer types is between zero and one also does not ma
