# Supplementary material for: Optimal three-part tariff pricing and marketing strategies for consumer overconfidence
Source: PLoS One. 2024 Nov 20;19(11):e0297819. doi: 10.1371/journal.pone.0297819 (PMC11578462; doi:10.1371/journal.pone.0297819)
Supplement: Attachment — Submitted filename: Response to Reviewers.docx [file pone.0297819.s004.docx]

**PLOS ONE**

**Manuscript ID**: **PONE-D-22-14682**

**Title**: Optimal three-part tariff pricing and marketing strategies for consumer overconfidence

We are pleased to have been given this opportunity to revise our manuscript. We carefully considered the comments of the editor and reviewers and made the corresponding changes. In this document, we explain how we revised our paper based on the comments and recommendations. We wish to extend our appreciation to the editor and reviewers for taking the time and making an effort to provide insightful guidance. We hope that our revisions improved our paper such that the editor and reviewers will deem it worthy of publication in *PLOS One*. We present our detailed responses to the comments below.

**Academic Editor**

**Comments #1**

Note that this is a very risky resubmission. One reviewer recommends a major revision and the other rejection, with very solid arguments. Therefore you need to resubmit a significantly improved manuscript that carefully addresses all comments. I believe that you can do it if the model is carefully reformulated, making it elegant and simpler, following the reviewers recommendations. As it stands, you are over concluding while your results are very weak and easily questionable.

**Response to Comments #1**

We thank the academic editor for the comment and for giving us a chance to improve our paper. We agree with the academic editor that we may have over concluded our research, while our results may not be sufficiently strong and may be easily questionable. We acknowledge that the paper was not perfectly prepared, and we apologize for that. We considered and addressed all the comments from the academic editor and reviewers. Specifically, we tried to improve the manuscript and made several major improvements, summarized as follows:

1. We added some important information related to the assumptions and parameters used in the study, the parameters used in the genetic algorithm, and the limitations of the study.
2. We rewrote the parts that seemed vague in Section 2 and Section 2.1.
3. We revised and reformulated the analytical model in Section 2.2 to clarify that in the profit maximization model, the consumers are uniformly distributed between 0 and 1. Similarly, in the numerical analysis, the consumer types are also between 0 and 1.
4. We added an analysis of the results in Section 5.4 regarding the price discrimination capacity with respect to the analysis of the three-part tariff pricing menu.
5. We clearly highlighted our contributions and contrasted our approach from the work of other researchers. The main contribution of our study is different from that discussed by Grubb (2009) and Fibich et al. (2017), which focused mainly on the optimal three-part tariff. In our study, we not only consider the cognitive bias in the model formulation but also investigate the three-part tariff pricing menu. Moreover, our study successfully identifies key factors or parameters (i.e., overconfidence level, consumer preferences, consumers’ price sensitivity, and variable costs) and the interaction of the factors affecting the performance of different pricing structures.

**Comments #2**

You need to explain and justify your assumptions and evaluate the sensitivity and limitations of your results accordingly

**Response to Comments #2**

We are thankful for the academic editor’s comment. The academic editor’s point is valid. We agree that we should clearly explain and justify the assumptions we used in our study. We clarified the assumptions and included some additional information in the revised manuscript. We also highlighted the limitations of the results. Please see the revisions on pages 6-12 in Section 2 and on pages 36-37 in Section 6.

We also pointed out that the purpose of the simulation in our study is slightly different. The simulation in Sections 5.3 and 5.4 focuses on the experimental design that we use to investigate the effect of changes in some of the parameters on the optimal solution. In our case, we change and set some of the parameters (i.e., overconfidence level, mean of the preference parameter, variance of the preference parameter, variance of price sensitivity, and variable costs) to high and low, which reflect the market structures or consumer preferences in the market. We did try performing the same analysis using different values and ranges of the parameters, and obtained similar and consistent results. Since those results are not the most critical ones in discussing our findings, we thus do not include a separate subsection of sensitivity analysis for the results of the simulation.

**Comments #3**

In addition, I want you to consider the ethical implications of your work. How can the market use your results to improve consumer well-being instead of simply exploiting a cognitive bias for profit? Please discuss this in depth.

**Response to Comments #3**

We are thankful for the insightful comment and suggestion. We acknowledge that the implication of our study does not put all the ethical issues into consideration, and we use specific formulations that focus on profit optimization, which is one of the limitations of our study. However, our study has methodological implications that may be applied to a similar system or circumstance, in which the manager’s or service provider’s focus is different. In the real world, other issues may be considered in the implementation of such a pricing mechanism to improve consumers’ wellbeing.

From our study, we can learn that if we apply the methodology to other situations using other formulations for the objective function, such as trying to increase the total usage consumption or number of participants, in the future, then we can also design some mechanisms that can improve consumers’ wellbeing even when they are overconfident. In this situation, we might be able to better understand how to increase the benefits of society when consumer cognitive bias is taken into consideration. Even though we are unable to accomplish it at this time, our study provides directions to others and a basis for the modeling framework. We added a discussion on this information in Section 6 of the revised manuscript. Please see the corresponding changes on pages 36-37 in Section 6:

*This study’s focus on profit optimization rather than on ethical implications is one of its limitations. However, this study has methodological implications that may be applied to similar systems or situations, in which the manager’s focus is different. In the future, this methodology may be extended to consider additional factors, such as enhancing consumer wellbeing, increasing the total usage consumption, or boosting the level of user participation, even when the consumers are overconfident. In this case, we might be able to better understand how to increase the benefit of society when consumer cognitive bias is taken into consideration. Even though we are unable to accomplish it at this time, this study provides directions to others and may serve as a basis for the modeling framework.*

**Reviewer #1:**

This paper studies the benefits of three-part tariffs under demand uncertainty caused by overconfident consumers. The research question is interesting and results are reasonable and potentially useful in practice. I have several comments that may be useful for the authors to strengthen this paper’s exposition and contribution.

**Comments #1**

The demand function, especially the uncertainty component, should be clearly explained.

**Response to Comments #1**

We are thankful to you for pointing out this issue. We agree that the uncertainty component of the demand function should be explained clearly. We apologize for not clearly explaining the uncertainty component in our manuscript. We addressed the reviewer’s concern by adding a discussion to explain the uncertainty component of the demand function on pages 6 in Section 2 of the revised manuscript:

*We suppose that among several consumers, each is distinguished by the preference parameter* $O$ *(the preference of the original consumers), with a range of 0–1, and a high* $O$ *value suggests high preference for the service. In other words, if* $O_{2}>O_{1}$*, then the* $O_{2}$ *consumers’ demand curve is above or to the right of that of the* $O_{1}$ *consumers. We expect consumer utility to increase linearly with* $O$ *and the two demand curves to not intersect as a result. The probability density of the original consumer type* $(O)$ *is uniformly distributed, which represents the variability or diversity between the consumers’ preferences; therefore, its function is* $f\left( O \right)$*= 1 for* $0\leq O\leq1$*. In our approach, we consider a situation in which asymmetric information exists between a service provider and individual consumers. A monopolist service provider who is maximizing its profits is aware of the* $f(O)$ *distribution of all the consumers but uncertain about which consumers he/she is facing, because the preference of the consumers is randomly generated from the distribution. By contrast, the consumers will have superior information, because they are aware of their own demand functions.*

**Comments #2**

How the overconfident consumers behave should be justified. What is the impact on the firm’s profit?

**Response to Comments #2**

Thank you for calling our attention to this important point. We agree that we should justify the overconfident consumers’ behavior and its impact on a firm’s profit. We discussed this important information on page 3 in Section 1 of the revised manuscript:

*In terms of psychological behavior, overconfidence is one of the most prominent problems in decision making (e.g., 2,4,5). From the perspective of consumers’ behavior, overconfidence may lead to an overly optimistic view of their ability being greater than their objective (original) consumption (e.g., 1,5–9). For instance, an overconfident consumer may overestimate his/her likelihood of consuming his/her Internet access, with a large gigabyte capacity. Service providers thus have an incentive to complicate their pricing structures (e.g., provide several pricing plans) to induce different customers to reveal their preferences and increase the consumption on demanded service [8,10,11]. By creating complicated pricing structures, service providers can better understand the consumption patterns and behaviors of overconfident consumers and understand how and when different tariff structures should be adopted [1]. Hence, examining the impact of overconfident consumers has important implications for pricing design and service providers’ profits.*

**Comments #3**

Some mathematical formulas are too lengthy or tedious, without transition or comments, for example equations (15), (25) and others.

**Response to Comments #3**

We thank the reviewer for the comment. We agree that some of the formulas are lengthy or tedious. We acknowledge and apologize for this issue. We improved the mathematical formulations by adding comments or transitions and some intermediate results in the formulation for simplification. Please see the improved parts in Section 2.2 of the revised manuscript.

**Comments #4**

The comparative statics seem interesting. Can you derive similar analytical results, in addition to numerical study?

**Response to Comments #4**

We are thankful for the reviewer’s comment. Yes, we can and we did obtain similar analytical results. Nevertheless, we did not include the results in our analysis, as they would not be beneficial for generating management implications. As a result, in the second part of our study, we concentrated on the numerical analysis and identified managerial implications that would be helpful to practitioners. The examples below are additional information to demonstrate the analytical results we obtained that are not included in the manuscript.

1. To determine the impact of variable costs $(mc)$ on profit$,$profitis partially differentiated into $mc$, which can be obtained by

$$\frac{\partial profit}{\partial mc}=\partial\frac{1}{25\lambda^{3}} (-\mathrm{mc}^3 \lambda-\mathrm{mc}(3+26r+23r^2 ) \lambda^3+2\mathrm{mc}(-1+9r)\lambda\surd(\lambda^2 (\mathrm{mc}^2+2\mathrm{mc}(-1+9r)\lambda+(1+r)^2 \lambda^2 ) )+(1+r)^2 \lambda^2 ((1+r) \lambda^2+\surd(\lambda^2 (\mathrm{mc}^2+2\mathrm{mc}(-1+9r)\lambda+(1+r)^2 \lambda^2 ) ))+\mathrm{mc}^2 ((3+23r) \lambda^2+\surd(\lambda^2 (\mathrm{mc}^2+2\mathrm{mc}(-1+9r)\lambda+(1+r)^2 \lambda^2 ) ))) )/\partial mc$$

$$\frac{\partial profit}{\partial mc}=\frac{1}{25\lambda^{3}}\left( -\mathrm{mc}\left( 26+46r \right)\lambda^{3}+\frac{\mathrm{mc}\left( -1+9r \right)\lambda^{3}\left( 18\mathrm{mc}\lambda+2\left( 1+r \right)\lambda^{2} \right)}{\sqrt{\lambda^{2}\left( \mathrm{mc}^{2}+2\mathrm{mc}\left( -1+9r \right)\lambda+\left( 1+r \right)^{2}\lambda^{2} \right)}}+18\mathrm{mc}\lambda\sqrt{\lambda^{2}\left( \mathrm{mc}^{2}+2\mathrm{mc}\left( -1+9r \right)\lambda+\left( 1+r \right)^{2}\lambda^{2} \right)}+\left( 1+r \right)^{2}\lambda^{2}\left( \lambda^{2}+\frac{\lambda^{2}\left( 18\mathrm{mc}\lambda+2\left( 1+r \right)\lambda^{2} \right)}{2\sqrt{\lambda^{2}\left( \mathrm{mc}^{2}+2\mathrm{mc}\left( -1+9r \right)\lambda+\left( 1+r \right)^{2}\lambda^{2} \right)}} \right)+\mathrm{mc}^{2}\left( 23\lambda^{2}+\frac{\lambda^{2}\left( 18\mathrm{mc}\lambda+2\left( 1+r \right)\lambda^{2} \right)}{2\sqrt{\lambda^{2}\left( \mathrm{mc}^{2}+2\mathrm{mc}\left( -1+9r \right)\lambda+\left( 1+r \right)^{2}\lambda^{2} \right)}} \right)+2\left( 1+r \right)\lambda^{2}\left( \left( 1+r \right)\lambda^{2}+\sqrt{\lambda^{2}\left( \mathrm{mc}^{2}+2\mathrm{mc}\left( -1+9r \right)\lambda+\left( 1+r \right)^{2}\lambda^{2} \right)} \right) \right).$$

2. To determine whether the impact of the overconfidence level ($r$) on the profit is equal to the partial differential of the profit to $r$, which can be obtained by

$$\frac{\partial profit}{\partial r}=\partial\frac{1}{25\lambda^{3}}(-3\mathrm{mc}^2 \lambda-(3+26r+23r^2 ) \lambda^3+(\mathrm{mc}^2 \lambda^2 (2\mathrm{mc}+2(-1+9r)\lambda))/(2\surd(\lambda^2 (\mathrm{mc}^2+2\mathrm{mc}(-1+9r)\lambda+(1+r)^2 \lambda^2 ) ))+(\mathrm{mc}(-1+9r) \lambda^3 (2\mathrm{mc}+2(-1+9r)\lambda))/\surd(\lambda^2 (\mathrm{mc}^2+2\mathrm{mc}(-1+9r)\lambda+(1+r)^2 \lambda^2 ) )+((1+r)^2 \lambda^4 (2\mathrm{mc}+2(-1+9r)\lambda))/(2\surd(\lambda^2 (\mathrm{mc}^2+2\mathrm{mc}(-1+9r)\lambda+(1+r)^2 \lambda^2 ) ))+2(-1+9r)\lambda\surd(\lambda^2 (\mathrm{mc}^2+2\mathrm{mc}(-1+9r)\lambda+(1+r)^2 \lambda^2 ) )+2\mathrm{mc}((3+23r) \lambda^2+\surd(\lambda^2 (\mathrm{mc}^2+2\mathrm{mc}(-1+9r)\lambda+(1+r)^2 \lambda^2 ) ))))/\partial r$$

$$\frac{\partial profit}{\partial r}=\frac{1}{25\lambda^{3}}\left( -\mathrm{mc}\left( 26+46r \right)\lambda^{3}+\frac{\mathrm{mc}\left( -1+9r \right)\lambda^{3}\left( 18\mathrm{mc}\lambda+2\left( 1+r \right)\lambda^{2} \right)}{\sqrt{\lambda^{2}\left( \mathrm{mc}^{2}+2\mathrm{mc}\left( -1+9r \right)\lambda+\left( 1+r \right)^{2}\lambda^{2} \right)}}+18\mathrm{mc}\lambda\sqrt{\lambda^{2}\left( \mathrm{mc}^{2}+2\mathrm{mc}\left( -1+9r \right)\lambda+\left( 1+r \right)^{2}\lambda^{2} \right)}+\left( 1+r \right)^{2}\lambda^{2}\left( \lambda^{2}+\frac{\lambda^{2}\left( 18\mathrm{mc}\lambda+2\left( 1+r \right)\lambda^{2} \right)}{2\sqrt{\lambda^{2}\left( \mathrm{mc}^{2}+2\mathrm{mc}\left( -1+9r \right)\lambda+\left( 1+r \right)^{2}\lambda^{2} \right)}} \right)+\mathrm{mc}^{2}\left( 23\lambda^{2}+\frac{\lambda^{2}\left( 18\mathrm{mc}\lambda+2\left( 1+r \right)\lambda^{2} \right)}{2\sqrt{\lambda^{2}\left( \mathrm{mc}^{2}+2\mathrm{mc}\left( -1+9r \right)\lambda+\left( 1+r \right)^{2}\lambda^{2} \right)}} \right)+2\left( 1+r \right)\lambda^{2}\left( \left( 1+r \right)\lambda^{2}+\sqrt{\lambda^{2}\left( \mathrm{mc}^{2}+2\mathrm{mc}\left( -1+9r \right)\lambda+\left( 1+r \right)^{2}\lambda^{2} \right)} \right) \right).$$

Determining the positive or negative of the partial derivative from the examples above would be impossible. As the equation is quite complicated, obtaining the results directly from the equation would be impossible as well as identifying when some of the parameter changes will occur in our optimal solution.

**Comments #5**

Part 4 comes too late, and I suggest to discuss consumer overconfidence at the beginning.

**Response to Comments #5**

We are thankful for the suggestion. We moved part 4 to part 3. Please see the related revisions on pages 19-22 in the revised manuscript.

**Reviewer #2:**

This paper attempts to understand how firms may determine the optimal three-part tariff structure when facing overconfident consumers. Overconfident consumers tend to miscalculate their future consumption which may be used by firms to maximize profits. Firms may charge a higher fix cost for a specific number of unit allowance and a small usage fee for units consumed above that specified number, expecting overconfident consumers to pay the fix cost, and not consuming all the units’ allowance. In this context, the paper looks for the optimal parameters and the optimal menu of three parts tariff maximizing firms’ profits.

**Comments #1**

My first comment must be about how the paper has been written. The paper is very confusing to read, some parameters of the model are not well explained (for instance, the role of \theta_1 and \theta_2), and there seems to be a changed of variable at some point about the consumer types (being \theta at the beginning of section 2 and then becoming \theta+r in my interpretation). Many other typos of this type are found along the paper, but I will not mention all of them. I think is the role the authors to provide a paper more carefully written making the role of the reviewers easier

**Response to Comments #1**

We are thankful for the reviewers’ comment. We agree that the paper should be more carefully written, so it will be easier to read. Especially the discussion regarding the parameters of the model (for example, the role of \theta_1 and \theta_2), and the changed of preference parameter or consumer types (being \theta at the beginning of section 2 and then becoming \theta+r). We also thanks to reviewer for noting the other typos in the paper. We recognize our mistake in writing the paper, and we apologize for making a mistake like this. We have taken the comments on board by refining the paper. Please see the improvement parts in the revised manuscript. In particular we made some improvements as follows:

1. We have carefully improved in writing the paper and noting the notation in the paper, especially regarding the explanation of the parameter used in the study. Now, it might be easier to understand.
2. We have also discussed the reason why \theta or notation O in our manuscript at the beginning of section 2 becoming \theta+r or \O+r).
3. We tried to revise all the typos along the paper that the reviewer raised.

**Comments #2**

Other important issue to mention is that the paper does not really focus on the optimal three-part tariff structure because their solution depends on several specific parameters and assumptions. In the best-case scenario, they can find some local optimums but not the global optimum of the problem. This is even the case with the algorithm approach. This concern it's more problematic given the nonlinear nature of the profit functions they have.

**Response to Comments #2**

We thank the reviewer for highlighting this point. We agree that in our study, we made some assumptions to make the problem analytically tractable and ensure that we were focused on finding the optimal three-part tariff. We thank the reviewer for pointing out this limitation. Therefore, in our conclusion on pages 37 in Section 6, we added a discussion on this limitation, that is, the consumers are uniformly distributed within a closed interval and have the same level of overconfidence. In the future, we want to relax some of the assumptions by considering a flexible demand function with different levels of overconfidence.

For the parameters, other purposes exist. As the model is not analytically tractable, for the parameters, we try to understand how the optimal three-part tariff structure will change under different situational conditions. We intentionally change the parameters or use different parameter values to explore the properties of the optimal three-part tariff. We apologize for not explaining the procedure clearly and understand that it is our responsibility to provide a clear description. We discussed this issue on page 5 in Section 1.

We also agree that the algorithm cannot guarantee the global optimal solution, but we already carefully checked the efficiency of the algorithm. Although the results we obtain may not be the true global optimum, the algorithm should be able to achieve nearly global optimum results to help us derive some useful implications. We added this information on page 25 in Section 5.

However, for letting others know that it might also be essential directions for the future study, we included this information in the last pages of Section 6. Please see the revisions, as follows:

*Furthermore, this study derived from previous works on three-part tariff pricing. We assume that the consumers uniformly distributed within a closed interval have the same level of overconfidence. A highly flexible demand function with different levels of overconfidence will be beneficial and must be considered in future studies. We also view overconfidence as a type of irrational attribute to investigate the psychological aspects of behavior. Future studies can expand the research scope by considering other psychological behavior components that may have a strong impact on how three-part tariff pricing is implemented. Despite the fact that we already have procedures to make sure that genetic algorithms can achieve nearly optimal solution, it may still be possible to investigate more efficient ways of solving optimal solutions for this kind of problem in the future.*

**Comments #3**

These nonlinearities are also relevant for comparative static analysis where the authors use only two different values for the marginal cost and the consumer price sensitivity. The effect found cannot be generalize to a different range of numbers and the authors do not explain why the numbers selected are the most relevant in this context. In particular, the price sensitivity equal 1.

**Response to Comments #3**

We are grateful to the reviewer for pointing out this issue and note the reviewer’s comment. First, we must clarify that the term “comparative statics analysis” should be changed, because our procedure may seem less like a comparative statics analysis in economics and more like a numerical analysis for examining how changes in the parameters will affect the optimal solution. We made the corresponding changes in Section 4. Second, we appreciate the reviewer for pointing out the issue of nonlinearity, especially why we use only two levels of marginal costs and consumer price sensitivity, which may not be able to capture the nonlinearity effect of the lambda or marginal costs on the optimal solution.

We agree with the reviewer that investigating the nonlinear or higher-order effects of the lambda or marginal costs on the optimal solution is important, however, as discussed previously, the focus or key function of the numerical analysis is to understand how different tariff menus work under different market structures or consumer preferences. A two-level experiment design can better serve the purpose and help us investigate the impacts of the changes in some of the parameters with different values (i.e., overconfidence level, marginal costs, and consumer price sensitivity). We did pointed out that investigating the nonlinear or higher-order effects of the parameters on optimal solution should be an important direction for the future research. Please see the revisions on pages 36-37 in Sections 6:

*While this research presents the clear benefit of providing useful information into the design and implementation of three-part pricing systems when customers are overconfident, certain drawbacks are encountered. This study’s focus on profit optimization rather than on ethical implications is one of its limitations. However, this study has methodological implications that may be applied to similar systems or situations, in which the manager’s focus is different. In the future, this methodology may be extended to consider additional factors, such as enhancing consumer wellbeing, increasing the total usage consumption, or boosting the level of user participation, even when the consumers are overconfident. In this case, we might be able to better understand how to increase the benefit of society when consumer cognitive bias is taken into consideration. Even though we are unable to accomplish it at this time, this study provides directions to others and may serve as a basis for the modeling framework. In addition, this study succeeded in identifying the impacts of the changes in some of the parameters on the optimal solution, however, investigating the nonlinear or higher-order effects of the parameters on optimal solution should be an important direction for the future research.*

**Comments #4**

I am aware that the difficulties to solve these types of models open the door to this type analytical and numerical approaches, but they are usually a tool used to understand the role of a particular mechanism. However, the mechanism in your paper was already known and your contribution is to find the optimum three-part tariff. In my opinion, this methodology does not allow you to solve this problem.

**Response to Comments #4**

We thank the reviewer for pointing out this issue. We agree that other researchers proposed some approaches to analytically solve the optimal three-part tariff problem under certain assumptions (e.g., Grubb 2009; Fibich et al. 2017). However, in our study, we try a different approach. The main contribution of our study differs from that of previous studies, which paid attention mainly on the optimal three-part tariff. In our study, we not only consider the cognitive bias in the optimal three-part tariff but also investigate the three-part tariff pricing menu.

We acknowledge that this methodology is not perfect. However, the methods we employ can help us investigate the optimal three-part tariff pricing and achieve our desired implications. We added some discussion with respect to our study’s limitations, which we mentioned in our response to comment #2. We tried to highlight the significance of our study in a persuasive manner and added a discussion on the other studies. Please see the revisions on page 4 in Section 1 of the revised manuscript:

*The study on the optimal three-part tariff pricing has been merely investigated analytically in a few research, and the literature on three-part tariff pricing is mostly empirical or numerical (e.g.,16–20). Specifically, two studies conducted analytical analyses on the optimal three-part tariff optimization problem, namely, Grubb [21] and Fibich et al. [22]. For overconfident consumers, Grubb [21] investigated the optimal three-part tariff pricing by assuming that each customer has an estimated demand and actual demand and chooses a plan based on his/her estimated demand. The author demonstrated that when customers are overconfident, companies can make more money. Fibich et al. [22] considered a symmetric information between companies and consumers as well as a theoretical model in which a service provider offers services to risk-averse and rational consumers. The main contribution of our study is different from that of Grubb [21] and Fibich et al. [22], which focused mainly on the optimal three-part tariff. In our study, we also include cognitive bias in the model formulation and consider investigating the three-part tariff pricing menu.*

**Comments #5**

Respect to the assumptions of the model, I wonder why the authors assume that the level of overconfidence is the same for all consumers in the population. Moreover, they assume that there is no relationship between their types and their level of overconfidence. Of course, these assumptions help to maintain the uniform distribution of types and the tractability of the model, but it also limits its applications. Could the authors extend their analysis to other types of distributions?

**Response to Comments #5**

We thank the reviewer for providing this crucial comment. The reviewer is correct; in our study, we assume that the level of overconfidence is the same for all the consumers in the population, and no relationship exists between consumer types and level of overconfidence. We agree with the reviewer that the assumptions can help maintain the uniform distribution of the consumer types and tractability of the model. Although the assumptions are strong, we find that adopting the current formulation can provide useful and insightful results. However, we are aware that this issue is important and should be considered in future studies. As we mentioned in our response to comment #2, we added an explanation for this limitation on pages 37 in Section 6 of the revised manuscript:

*Furthermore, this study derived from previous works on three-part tariff pricing. We assume that the consumers uniformly distributed within a closed interval have the same level of overconfidence. A highly flexible demand function with different levels of overconfidence will be beneficial and must be considered in future studies. We also view overconfidence as a type of irrational attribute to investigate the psychological aspects of behavior. Future studies can expand the research scope by considering other psychological behavior components that may have a strong impact on how three-part tariff pricing is implemented. Despite the fact that we already have procedures to make sure that genetic algorithms can achieve nearly optimal solution, it may still be possible to investigate more efficient ways of solving optimal solutions for this kind of problem in the future.*

**Comments #6**

At the beginning, the model assumes that consumer types are uniformly distributed on the range between zero and one. However, in the profit maximization of the firm the consumers are distributed between \theta star and \theta upper bar. Similarly, in the numerical analysis consumer types are not between zero and one. The uniform distribution between zero and one make it easier to calculate the consumer surplus but then you cannot change the range of the distribution. The authors should explain better the reasons behind this change.

**Response to Comments #6**

We thank the reviewer for the comment and suggestion. The reviewer is correct that the model assumes that the consumer types are uniformly distributed within the range of 0–1. We apologize for our mistake in noting the range of the model. We revised and reformulated the analytical model in Section 2.2. In formulating the consumer decision and profit optimization, we use the original consumer type which is uninformly distributed with range from 0 to 1. We integrate the function from O* to 1 for those consumers that really subscribe the service. Specifically, O* represents the original consumer type and differentiates whether or not a consumer will subscribe to a service. It is also a threshold that can determine the minimum value of the type of consumer who will subscribe to a service. A consumer will subscribe to a service if his/her type is higher than O* but will not subscribe to a service if his/her type is lower than O*. Similarly, the consumer types in the numerical analysis should range from 0 to 1. We appreciate the reviewer’s suggestion that we should clarify this subject thoroughly. We revised the analytical model and changed some of the notations to make our paper consistent. Please see the revisions in Section 2.2 and Section 3 of the revised manuscript.

**Comments #7**

Respect to the analysis of the number three-part tariffs in the menu offered by the firm, it is not surprising that you find that a menu with more options dominates the others. Basically, more pricing options allow you for a better discrimination of the consumers and therefore higher profit. Considering this a more interesting analysis will be to determine when this discrimination capacity matters the most.

**Response to Comments #7**

Thank you for calling our attention to this point. We agree that an interesting analysis of the three-part tariff menu’s capacity to provide discriminant prices for inducing consumers with different preferences is important, especially when it matters most. We focused on this issue and clearly addressed the question raised by the reviewer. We made some changes and provided an in-depth discussion on the issue. Please see the revisions on pages 31-32 in Section 5 of the revised manuscript:

*Specifically, the results in Table 8 show that the level of consumers’ overconfidence is a major factor influencing profits (i.e., whether or not consumers have a high level of overconfidence for a service). This factor accounts for roughly 70% of the variance in profitability. Given that this finding on the main effect of consumers’ overconfidence is completely consistent with our expectations, a service provider can earn increased profits (or producer surplus) by selling a service to customers with a high overconfidence level. Fig. 3 shows that as the level of consumers’ overconfidence increases, profit increases, and vice versa. The profitability of a service provider increases by 65.52% when consumers’ overconfidence level is high.*

*In particular, the nonparallel lines in Fig. 3 show that when the overconfidence level is low, providing multiple three-part tariff structures can achieve better price discrimination and will have a significant impact on determining how much profits a service provider can earn, as the distance between the three lines in the figure is larger when the overconfidence level is low than when it is high. Single three-part tariffs can lead to low profits, but tariff structures that involve two-option or three-option three-part tariffs can lead to relatively high profits, and a service provider’s profitability will decrease by approximately 13.27% when only single three-part tariff is used. By contrast, the difference in profits among various tariff structures will be relatively small when the overconfidence level is high, and a service provider’s profitability will decrease by approximately 2.51% when only single or two-option three-part tariffs are used.*

**Comments #8**

The authors use the demand function coming from Tirole (1988) and they argue that they’re reasonable assumptions for the appropriateness of this demand function in terms of utility theory. I think the authors should discuss those assumptions in the paper and to discuss how appropriate is this function to study overconfidence.

**Response to Comments #8**

We thank the reviewer for the comment and suggestion. The reviewer is correct; we use a specific demand function from Tirole (1988) and employ a reasonable assumption for the appropriateness of the demand function in terms of utility theory. We agree with the reviewer that we should discuss our assumptions and the appropriateness of the function to examine overconfidence. Originally, the demand function is appropriate for a regular case, in which a consumer is not overconfident, such as in the research of Jang and Kwon (2014). In our study, we attempt to establish a specific type of overconfidence, or overestimation, using a similar demand function. Although the demand function may not completely reflect the behavior of real-world overconfident consumers, the formulation can be helpful to investigate the problem and obtain some important insights. We discussed the assumptions related to the demand function as well as the appropriateness of the demand function to examine overconfidence. Please see the revisions on pages 6-8 in Section 2 of the revised manuscript:

*Following Tirole [23], we build a simple consumer demand function, where demand is assumed to be a linear function (with a non-negative intercept and constant negative slope) of price and independent of time. This function is extensively used in economics and in the operations management literature (see [16,17,20,24–27] ). In particular, the linear demand function can be justified in terms of utility theory. In other words, a demand function can be derived from a utility function together with a budget constraint. Thus, with reasonable assumptions of the consumer’s behaviour in real world practices that under a given budget they will maximize the utility function that is quadratic in the usage consumption, the first order conditions for the consumer problem do result in linear demand when the utility function is strictly concave in the quantities consumed. Since this utility function is relatively flexible and can be applied to many real-world cases, we can justify the appropriateness of the linear demand function in terms of utility theory and considerably simplify the analytical model in this study. The linear demand function is defined by Equation (1):*

$q= O-\frac{1}{\lambda}p ,$*(1)*

*where* $q$ *is the quantity demanded by the consumers;* $p$ *is the price of the goods or services; the constant* $\lambda$ *(or* $\frac{1}{\lambda}$*) is the slope of the demand curve, which shows the sensitivity of the quantity demanded for the product or service price; and the intercept* $O$ *represents the original consumer type, with varying preferences and used to formulate the aggregate effect of the non-price factors on the quantity demanded. For a given price, the quantity demanded is high when preference* $O$ *is high.*

*In the following section, we use the basic model of Jang and Kwon [28], first assuming that the density function of the original consumer type obeys a uniform distribution, with a probability density function* $f\left( O \right)=1.$ *We assume that the cost function C(∙) has low constant marginal costs, because variable costs in the telecommunications industry are typically low. Then,* $C\left( Q \right)=mc$*.* $Q$*, where*$Q$ *is the total consumption, and* $mc$ *is the constant marginal costs.*

*In this study, we define consumers’ overconfidence using the definition in Dellavigna and Malmendier [10], that is, the measure of consumers’ overconfidence is given by the difference between the estimated (forecasted) and original consumption. In this situation, the overconfident consumers overestimate their future demand and ability to take advantage of their consumption of the demanded service as well as the units consumed. From the service provider’s perspective, usage overestimation is equivalent to an upward shift in demand. This condition is illustrated in Fig. 1, given that among the overconfident consumers, the demand curve shifts upward by*$(r)$*, which is the amount that the consumers overestimate their unit consumption from the original consumer preference* $(O)$*. Thus, we use the following expression to explain the demand of the overconfident consumers:*

$q=\left( O+r \right)-\frac{1}{\lambda}p$*.	(2)*

*The notation* $O+r$*is a special way to define the consumers’ overconfidence, which we consider as a type of overestimation and means that after* $O+r ,$*the consumers will become overconfident. Notation* $O$ *denotes a preference parameter of the original consumer type. The constant* $r$ *is a consumer’s level of overconfidence that he/she will consume a number of units. We assume that the overconfidence level* $(r)$ *is the same for all the consumers. In the next subsection, we analyze consumer purchase decisions and the profit-maximizing model for service providers from the perspective of the original consumer type.*

**Comments #9**

More explanation is needed in the genetic algorithm. What determines the selection of individuals in each iteration? Do you use any measure of fitness in this case, or everything is random from the original population distribution?

**Response to Comments #9**

Thank you for calling our attention to this point. We agree with the reviewer and apologize for not clearly explaining the information about the selection process and how we measure the fitness value. We addressed all the questions raised by the reviewer and we not only discuss the selection process but also the parameters that can be used to evaluate the fitness value. Please see the revisions on pages 25-27 in Section 5 of the revised manuscript:

*The natural selection process described by Holland [30] in biology served as the basis for the development of the genetic algorithm (GA), which is a metaheuristic technique renowned for its ability to handle large search areas. Implementing a real-valued GA in many systems would be straightforward [31,32]. A population is first generated by this algorithm at random. A group of individual solutions can be used to characterize the population (chromosomes). In the selection process, a small number of solutions are chosen by the algorithm from the current population to act as parents to create a new generation based on the fitness value of the parents. A chromosome is chosen at random from the population, with the probability determined by the fitness function. Genetic processes such as crossover and mutation are responsible for producing a new generation. When the termination condition is satisfied and the best chromosome from the most recent generation is offered as the final solution, the new generations will be assessed [33]. The number of generations, population size, crossover rate, and mutation rate are some of the parameters that GA programs typically include to show the four primary genetic operators linked with the algorithm [34]. The GA produces an efficient, optimal, or a nearly optimal solution depending on the parameters. The four important GA parameters we employ in our study are summarized below.*

1. *Generations: The total number of cycles necessary for a generation to finish; the termination criterion in this study is based on the maximum generation, which is set to 1,000 generations. The individual with the best fitness value across 1,000 generations is considered to be the optimal solution.*
2. *Population size: 50 chromosomes are randomly selected as the population of this study. A population’s size reveals details about its structure. According to De Jong and Spears[35], the ideal population size should fall between 50 and 100.*
3. *Crossover rate: To create a new offspring, the parents, who will be used in the crossover, are chosen. In the chromosome, where the exchange of the genetic material from the parents occurs, a random gene is chosen to implement the crossover. Then, a new offspring is produced by the crossover, depending on the chosen exchange point, with specific parent components. We employ a real-value crossover in this study to choose the point at random, which then transfers the necessary components from the two parents to produce an offspring. The offspring can be determined as follows:*

$$o_{i}=\alpha\cdot x_{i}+\left( 1-\alpha\right)\cdot y_{i},$$

*(35)*

*where* $\alpha$ *is a random number with a uniform distribution between 0 and 1, and* $x_{i}$ *and* $y_{i}$ *are the two chosen gene-pool chromosomes.*

4. *Mutation rate: Mutation often occurs after the completion of the crossover. The operator generates new adaptive solutions and helps prevent local optima by applying the modifications arbitrarily to one or more “genes” to create new offspring. Similar to a crossover, real-value mutation [36] is a method for producing new offspring from parents. The following can be used to determine the mutation rate:*

$$z_{i}=x_{i}+sign\cdot r_{i}\cdot\delta.$$

*(36)*

$Sign\in\{+1, -1\}$ *is chosen with equal probability,* $r_{i}=\rho\left( {range}_{i} \right), \rho\in\left[ 0.1, 0.5 \right].$*The range ratio (*$\rho$*), which is also known as the variable range ratio, is related to the maximum step that a mutation is permitted to produce;* ${range}_{i}$ *is the range of variable* $x_{i}$*; and* $t$*he parameter* $\delta$ *indicates the extent to which offspring can be produced from the parents and is expressed as follows:*

$\delta=2^{-k.u}$*,*

*(37)*

*where* $k\in N^{+}$*, and* $u\in[0,1]$*, with uniform probability. The mutation precision level necessary to reach the optimum depends on parameter* $k$*. However, in practice, the value of k is determined by the expected value of the mutation steps, and the higher the value of*$k$*, the more precise the resulting mutation operator. The GA operators employed in this investigation are described in detail below. To update the solution, we set*$\rho$ *to 0.1, signifying a low mean mutation step. Moreover, we set* $k$ *to 10, which complements the approach and is known to have a significantly strong influence on performance.*

**Comments #10**

It is important to differentiate throughout the paper between the consumer surplus and expected consumer surplus, based on the difference between consumption and expected consumption that the authors do not make.

**Response to Comments #10**

We thank the reviewer for providing this important suggestion. We agree that we must differentiate between consumer surplus and expected consumer surplus based on the difference between consumption and expected consumption. In this study, each individual consumer will make a decision based on their own demand function. As a result, the consumer is experiencing consumer surplus rather than expected consumer surplus and no expected consumption.

However, as the service provider must deal with the distribution of different type of consumers, we discuss the expected profit for the service provider. In that situation, we have the expected consumption rather than the consumption.

Thus, we have the expected consumption but not the expected consumer surplus. The expected consumption for profit maximization is the service provider’s decision, facing different types of consumers and needing to consider the distribution and find the optimal expected consumption. In this case, we try to maximize the expected profit and expected consumption. We made improvements in the paper by differentiating between consumer surplus and expected consumption. Please see the revisions in the manuscript.

**Comments #11**

In section 2.1, you include a double characterization of the consumers. First, you mentioned that there are two types of individuals, \theta_1 and \theta_2, but then you use these two types to characterize regions of consumers. The explanation is confusing, and you should differentiate the role of these categories better.

**Response to Comments #11**

Thank you for calling our attention to this point. We agree that we should clearly explain and differentiate between the role of \theta_1 and \theta_2 (in the manuscript, we use \O_1 and \O_2), especially in Section 2.1. We apologize for the confusion. We improved the explanation on the role of \O_1 and \O_2. We hope that the revised paragraph is easy to understand. Please see the improvements in the revised manuscript:

*To formulate the consumer purchase decision and profit maximization, we use the original consumer type. We assume that the consumers face a monopolistic service provider offering a three-part tariff scheme comprising a fixed fee* $(T)$*, unit price* $(p_{0})$*, and usage allowance* $(q_{0})$ *under a fixed fee. We first define the consumers’ willingness to pay and decision to subscribe to a service, amount of consumer surplus, and quantity or units of service consumed. Below, we present two distinct approaches for determining willingness to pay for consuming* $q$ *units of service, which may be specified by rewriting the customers’ demand function as* $p=\left[ \left( O+r \right)-q \right]\lambda$*. For consumers with preference parameter* $(O+r)$*, willingness to pay can be defined as follows:*

I. *When* $q \geq O+r$*,*

$$WTP\left( q \right)= \int_{0}^{O+r} \lambda\left[ \left( O+r \right)-q \right]dq$$

$=\frac{1}{2}\lambda\left( O+r \right)^{2}$*.	(3)*

II. *When* $q < O+r$*,*

$$WTP\left( q \right)= \int_{0}^{q} \lambda\left[ \left( O+r \right)-q \right]dq$$

$=\left( O+r \right)\lambda q-\frac{1}{2}\lambda q^{2}.$	*(4)*

*When formulating willingness to pay, we assume that the probability density of the original consumer type* $(O)$ *is uniformly distributed and can be a continuous uniform variable with a range of 0–1. However, we assume that* $\lambda$ *is the same for all the consumers. Then, we categorize the consumers into three groups under a single three-part tariff based on the consumer indexes, as shown in Table 1.*

***Table 1. The consumer indexes***

| *Index* | *Characteristic* | *Value* |
| --- | --- | --- |
| $O_{1}$ | $O_{1}$ *consumes* $q_{0}$ *when the fixed fee and unit price are 0* | $O_{1}=q_{0}$ |
| $O_{2}$ | $O_{2}$*consumes* $q_{0}$ *when the fixed fee is 0 but the unit price is* $p_{0}$ | $O_{2}{= q}_{0}+ \frac{1}{\lambda}p_{0}$ |

*The* $O_{1}$*consumers consume* $q_{0}$ *when the fixed fee and unit price are 0, and the* $O_{2}$*consumers consume* $q_{0}$ *when the fixed fee is 0 but the unit price is* $p_{0}$*. Under a single three-part tariff, the monopolistic service provider has three endogenous variables. The amount of the fixed fee* $(T)$ *will determine whether or not the consumers will subscribe to the service and extract the surplus. If the fixed fee* $(T)$ *is greater than the surplus obtained from the consumers’ unit consumption, then the consumers will not subscribe to the service, and the service provider will maximize profits by adjusting the fixed fee* $(T)$*, unit price* $(p_{0})$*, and usage allowance* $\left( q_{0} \right).$

*Fig. 1 shows the three groups of consumers. Fig. 1(b) reveals that the first consumer type within the interval* $(0, O_{1})$ *consumes less than the usage allowance (*$q_{0}$*). Even when the unit price is 0, and with the option of consuming the entire usage allowance, such consumers will prefer to consume less than the usage allowance (*$q_{0})$*. Fig. 1(c) presents the second consumer type, with a preference ranging from (*$O_{1}, O_{2}-r$*), who consumes the exact amount of usage allowance when choosing to subscribe. Such consumers do not consume more than the usage allowance, because the obtained surplus is less than the unit price. Fig. 1(d) shows the third consumer type, with a preference ranging from (*$O_{2}-r, 1$*), who consumes more than the allowance given, as the additional consumer surplus obtained when using one unit of service is higher than the unit price. Even when their consumption is higher than the allowance, such consumers will continue to consume until the marginal benefit is equal to the unit price.*

**Comments #12**

The last paragraph section 2.1 we shall have \theta+r in many places, it looks like a mistake. But an alternative explanation is that they are using \theta as \theta+r which is confusing giving his presentation of the model in Section 2.

**Response to Comments #12**

We thank the reviewer for correcting this point. We agree that there are several \theta+r in the last paragraph of Section 2.1, and we use \theta+r as \theta. We apologize for including inappropriate notations and for the confusion in our presentation of the model in Section 2. We tried to improve this issue and the paper’s consistency. Please see the improvements in the revised manuscript:

*Willingness to pay or the gross consumer surplus can be used to formulate the model of whether or not the consumers will purchase a service and the amount of service units that will be consumed, depending on the three-part tariff offered. We let GCS (*$O)$ *be the function of the gross consumer surplus, with the overconfidence preference represented by the shaded area in Fig. 1. Specifically,* $CS\left( O_{1} \right)= \frac{1}{2}\lambda\left( O_{1}+r \right)^{2}$ *is related to Equation (3) of willingness to pay when the overconfident consumer (*$O+r$*) preference is less than the allowance* $q_{0}$*. In addition,* $CS\left( O_{2} \right)= \left( O_{2}+r \right)\lambda q-\frac{1}{2}\lambda\left( O_{1} \right)^{2}$ *is related to Equation (4) of willingness to pay when the overconfident consumer (*$O+r$*) preference is larger than the allowance* $q_{0}.$ *As it is known, whether or not a special type of consumer will subscribe to the service depends on the fixed fee* $(T)$*. Henceforth,* $O^{*}$ *will represent the original consumer type, which is indifferent about subscribing or not subscribing to the service. When* $T$ *is less than* $CS\left( O_{1} \right)$*, the* $O_{1}$ *consumers will subscribe to the service, and the original consumer type* $O^{*}$ *will be less than* $O_{1}.$ *We divide all the consumers subscribing to the service into three parts. First, the consumers within the interval* $\left( O^{*}, O_{1} \right)$*consume* $O-r$*, that is, the quantity when the unit price is equal to 0. Second, the consumers within the interval* ${(O}_{1}, O_{2}-r)$ *consume the exact usage allowance* $q_{0}$*. Finally, the consumers within the interval* ${(O}_{2}-r, 1)$ *consume* $O+r-\frac{1}{\lambda}p_{0}$*. When* $T$ *is between* $CS\left( O_{1} \right)$ *and* $CS\left( O_{2} \right)$*, the* $O_{1}$ *consumers will not subscribe to the service, and the original consumer type* $O^{*}$*will be within the interval* ${(O}_{1}, O_{2}-r)$*. Then, we divide the consumers into two parts. First, the consumers within the interval* ${(O}_{1}, O_{2}-r)$ *consume the exact amount of allowance* $q_{0}$*. Second, the consumers within the interval* ${(O}_{2}-r, 1)$ *consume* $O+r-\frac{1}{\lambda}p_{0}$*. Theoretically, we do not need to consider the case in which* $T$ *is larger than* $CS\left( O_{2} \right)$*, because the consumers will not consume any service unit if the fixed fee* $T$ *is greater than the consumer surplus.*

**Comments #13**

In the parametrization of the model, which are reasonable levels of overconfidence and why?

**Response to Comments #13**

Thank you very much for the comment. The reviewer is correct; we did not include detailed information on the parameterization of the model, including the reasonable level when considering overconfidence. In the parameterization of the model, the level of overconfidence we assumed is between 0.1 and 0.5. This range is purposely used to conduct the numerical analysis that reflect a reasonable range that can be used in practical situations. Specifically, we set five levels of overconfidence : very low, low, medium, high, very high, using 0.1, 0.2, 0.3, 0.4, 0.5 as the cutoff points, respectively. Several previous empirical studies have used similar values to measure the level of overconfidence. For example, Grubb (2009) assumes that a low level of overcofidence is 0.25 and a high level of overconfidence is 0.75. Similarly, Campbell et al. (2011) defined three levels of consumer overconfidence: low, medium, and high, using 0.3, 0.67, and 1 as cut points, respectively. Based on the numerical analysis and empirical evidence, it seems that the range we considered in this study is not too far from the range used in other studies and it could be a reasonable level to apply in real-world cases. We added relevant information on the parameterization of the overconfidence level. Please see the improvements in Section 4 of the revised manuscript.

*We also examine diverse estimations of the overconfidence level* $(OL)$ *for the optimal solutions, and the reasonable level of overconfidence is between 0.1 and 0.5. Specifically, we set five levels of overconfidence, that is,* $OL=$ *0.1 for very low,* $OL=$ *0.2 for low,* $OL=$ *0.3 for medium,* $OL=$ *0.4 for high, and* $OL=$ *0.5 for very high. When considering the reasonable range of the overconfidence level, several previous studies considered using the similar values (see, for example, Grubb [21] and Campbell et al. [29]. Figs. 2.1–2.4 show that an increase in the overconfidence level (*$OL)$ *will cause an increase in all the endogenous variables, such as the fixed fee* $(T)$*, unit allowance provided under the fixed fee* $(q_{0})$*, unit price* $\left( p_{0} \right),$ *and expected profits* $(\pi)$*. When the overconfidence level* $(OL)$ *is high, the fixed fee* $(T)$ *and unit allowance provided under the fixed fee* ${(q}_{0})$ *will increase to mitigate costs and increase profits, and the charge per unit* ${(p}_{0})$ *will also increase, accordingly.*

**Comments #14**

Table 2 and figure 2 show the same information they should move one of them to the appendix.

**Response to Comments #14**

We are very thankful for your suggestion. We moved Table 1 to the Appendix. Please see the corresponding changes in the revised manuscript.

**Comments #15**

A sensibility analysis is needed in the simulation respect to the main parameters to determine in what range your conclusions are valid.

**Response to Comments #15**

We thank the reviewer for pointing out that a sensibility analysis (sensitivity analysis) is needed in the simulation with respect to the main parameters to determine the range within which our conclusions are valid. Although our study is a simulation study, it differs slightly from regular simulation studies. We try to conduct an experiment using the simulated data, and the simulation is similar to the sampling from the distribution. Other studies (i.e., Schlereth et al. 2010) also used a simplified distribution; for example, they used a triangle distribution to conduct experiments. Although our distribution is simple and may not reflect real-world situations completely, the distribution type has satisfactory properties and can help us investigate the issues.

In particular, the simulation in Sections 5.3 and 5.4 focuses on the experimental design that we use to investigate the effect of changes in some of the parameters on the optimal solution. In our case, we change and set some of the parameters (i.e., overconfidence level, mean of the preference parameter, variance of the preference parameter, variance of price sensitivity, and variable costs) to identify the key factors and investigating the main effect of those factors that might affect the optimal solution and the performance of different pricing structures. We did try performing the same analysis using different values and ranges of the parameters, and obtained similar and consistent results. Since those results are not the most critical ones in discussing our findings, we thus do not include a separate subsection of sensitivity analysis for the results of the simulation.

**Comments #16**

In section 2.2, the firm should not maximize respect to marginal costs that's something that you should take as given.

**Response to Comments #16**

Thank you very much for calling our attention to this point. We agree that in Section 2.2, the firm should not maximize marginal costs for each unit, which we should have taken as a given. Therefore, we rewrote the specific sentence to clarify the idea. Please see the revisions on pages 12-13 in Section 2.2, as follows:

*In this section, we present the formulation of the optimization problem of the monopolistic service provider. If only a single three-part tariff pricing scheme is considered, then the profit will be composed of two elements: the fixed fee paid by the consumers subscribing to the service* $(T$*) and the price paid by the consumers for purchasing units of service exceeding the basic amount* $(p_{0})$*. We let* $N$ *be the total number of consumers subscribing to the service, which can be obtained by integrating the density function* $f\left( O \right)$ *from* $O^{*}$ *to* $1$*. Thus, N is equal to* $\int_{O^{*}}^{1} f\left( O \right)ⅆO=\int_{O^{*}}^{1} ⅆO=1 -O^{*}$*. We let* $Q$ *be the expected unit consumption for all the consumers and* $Qe$ *be the expected unit consumption in excess of the allowance, which are both affected by the amount of the fixed fee* $(T$*). We construct the expected profit function of the monopolist service provider as follows:*

$\Pi=T\cdot N{+ p}_{0}\cdot Qe-mc\cdot Q$ *.	(5)*

*The objective function of Equation (5) is to maximize the expected total profits* $\Pi$*. The expected total profits is the sum of the profits from the total number of consumers (N) subscribing to the service by charged the fixed fee* $\left( T \right)$ *and the usage price for purchasing units of service exceeding the basic amount* $p_{0}\cdot Qe$ *minus the marginal costs of the expected total consumption* $mc\cdot Q$*. The decision variables of this optimization problem are* ${T, p}_{0}, and q_{0},$*which represent the price rate of the fixed fee, usage price, and allowance, respectively.*
