# Supplementary material for: Optimal three-part tariff pricing and marketing strategies for consumer overconfidence
Source: PLoS One. 2024 Nov 20;19(11):e0297819. doi: 10.1371/journal.pone.0297819 (PMC11578462; doi:10.1371/journal.pone.0297819)
Supplement: Attachment — Submitted filename: Response to Reviewers.docx [file pone.0297819.s005.docx]

**PLOS ONE**

**Manuscript ID**: **PONE-D-22-14682R1**

**Title**: Optimal three-part tariff pricing and marketing strategies for consumer overconfidence

We are pleased to have been given another opportunity to revise our manuscript. We carefully considered the feedback provided by the editor and reviewers and made the necessary changes accordingly. In this document, we explain how we revised our paper based on the comments and recommendations. We would like to extend our sincere appreciation to the editor and reviewers for their time and effort in offering us insightful guidance. We hope that our revisions improved our paper to the extent that the editor and reviewers will deem it worthy of publication in *PLOS One*. We present our detailed responses to the comments below.

**Academic Editor**

Thank you for submitting your manuscript to PLOS ONE. After careful consideration, we feel that it has merit but does not fully meet PLOS ONE’s publication criteria as it currently stands. Therefore, we invite you to submit a revised version of the manuscript that addresses the points raised during the review process.

As you can see, one reviewer recommends accepting the paper but the other rejecting it. I think you have done a remarkable job improving the paper, but the remaining concerns must be addressed. Reviewer 2 offers very precise feedback on what is still missing. I want to highlight the importance of showing that your results are not a trivial artifact of the model construction, but a true insight. That will be the key for me to make a final decision in the next round. The other comments are very doable.

**Response to Academic Editor**

We extend our gratitude to the academic editor for the comment and for offering us another opportunity to refine our manuscript. We acknowledge the valuable suggestion put forth by the academic editor. Specifically, we undertook additional analyses and presented more robust arguments in both our response and the revised manuscript. These analyses and discussions aim to demonstrate that the results of the current study are not mere artifacts of model construction; instead, they signify genuine insights. We have thoroughly considered and addressed the comments raised by Reviewer #2. A set of detailed responses to these comments can be found below.

**Reviewer #1:**

In the previous round, I provided 5 comments on the modeling, assumption, technical results and writing. In this round, the authors addressed all my comments very satisfactorily. The detailed changes are well documented in the response letter. Therefore, I have no additional comment and am happy to recommend to accept this manuscript.

**Response to Reviewer #1**

Thank you for taking the time to review our manuscript and for offering us your valuable feedback. We are delighted to learn that you deem our responses to your comments satisfactory and that no further comments have been raised. Your endorsement of manuscript acceptance is greatly appreciated, and we extend our gratitude for your support throughout the review process. Once again, thank you for your time and consideration.

**Reviewer #2:**

**Comments #1 (General Comment)**

Thank you for your attention to several of my comments in the previous round. You have improved the clarity of the paper in many of the sections, but there are still different issues that could be better explained. In this revised manuscript, the introduction and numerical analysis are much clearer, but the model needs more work. In any case, my main concern now is with the contribution of the paper. My comments to this version of the paper are the following:

**Response to Comments #1**

Thank you for dedicating time to reviewing our revised manuscript and for sharing your feedback. We are glad to know that you have found improvements in the clarity of the introduction and numerical analysis sections compared to the previous version. Your comments on the model are definitely valued, and we are actively considering them as we proceed with this round of revision. Below, you will find comprehensive responses addressing each specific comment you have raised.

**Comments #1a**

Contribution: Citing the authors: “The main contribution of our study differs from that of previous studies, which paid attention mainly on the optimal three-part tariff. In our study, we not only consider the cognitive bias in the optimal three-part tariff but also investigate the three-part tariff pricing menu.” In the paper, the authors showed that a three-part tariff menu with more tariffs plans dominate one when less tariff plans for all the different parameters on their numerical exercise comparing menus with three, two and one tariff. As I mentioned in my previous review in comment 7, this is not a surprising result because more tariff plans allow the firm to discriminate better. A higher ability to discriminate among clients helps the firm to increase its profits.

Moreover, the results of the experiment in section 5 shows that the menu is more profitable and increase participation the smaller the general over-confidence level. At a first glance, this result seems really interesting, but my intuition is it comes actually from the model construction. A lower over-confidence level implies we have two qualitatively different type of clients as shown in panel b and c in Figure 1. The clients in panel d are qualitatively equal to those on panel c. As a consequence, the advantages of the discrimination possibilities imply higher profits and user participation as the more qualitatively different are the clients of the firm. Then, the novelty of the contribution of the paper is not very convincing for me and I do not know if it is enough to grant a publication in the PLOS One journal.

**Response to Comments #1a**

Thank you for providing your valuable feedback on our manuscript. We really appreciate your comprehensive review and constructive comments. Firstly, we wish to sincerely apologize to the reviewer for not sufficiently conducting analyses and providing suitable interpretations to showcase the novelty and contribution of our current study. To rectify this, and in order to better emphasize the insightful findings and the value of our research, we have conducted a significantly more comprehensive simulation experiment and analysis. This has led to substantial revisions in Sections 5.3 and 5.4.

In particular, we have adjusted the parameter settings (while also relaxing a strong assumption employed in the earlier simulation) to ensure they more effectively aid in deriving actionable managerial insights. Additionally, our experimental design now includes a two-part tariff pricing structure, allowing us to compare it with the more intricate tariff structures. By examining how service providers design both the simpler two-part tariff and the more complex three-part tariff when faced with consumers exhibiting varying levels of overconfidence, we can derive improved managerial implications.

Furthermore, we have rectified minor errors that were present in our prior analysis, specifically within the experimental section of Section 5.4. These inaccuracies were due to our oversight in updating the model formulation for a portion of the experiment. Through supplemental analyses, discussions, and the correction of these errors, we believe that the current version of the manuscript effectively addresses the reviewer’s aforementioned comment.

Additionally, we have summarized the major changes implemented, providing justifications that aid in a better understanding of the reasons behind these modifications. This aims to assist reviewers in comprehending the rationale for our adjustments.

1. To enhance readability and establish a clear logical progression, as well as to offer sound justifications for our chosen experimental parameters, we have undertaken significant revisions of the initial paragraphs of Section 5.3. These revisions provide explicit explanations and rationales for our parameter selections within the experiment. Notably, we also elucidate the rationale for our chosen distribution in the simulation and elucidate its associated advantages. This elucidation is anticipated to enhance readers' comprehension of our experimental design of the numerical analysis.
2. In our prior analysis, a strong assumption was employed, presupposing that the levels of consumer overconfidence are the same. In the present analysis, to demonstrate the robustness of our findings, we have transformed this assumption. We now model consumer overconfidence levels as a distribution, accounting for heterogeneity. This approach captures the subtleties of consumer behavior more effectively and presents a closer approximation of real-world market dynamics.
3. Our experimental framework now integrates a two-part tariff pricing structure, serving as a point of comparison with more intricate tariff arrangements. While the two-part tariff structure involves one less component than the three-part tariff, equivalence between these two structures holds when consumer demand meets some general conditions (see, for example, the non-decreasing price elasticity (NPE) condition discussed in Bhargava and Gangwar (2018)). Hence, by examining the strategic choices of service providers when confronted with varying degrees of consumer overconfidence, we can discern distinctions between pricing structures. The inclusion of the two-part tariff enriches our analysis thus aids in extracting pertinent managerial insights.
4. A comprehensive re-evaluation of the entire experiment has been conducted, accompanied by substantial rewrites in the discussion of outcomes in Sections 5.3 and 5.4. We have expounded extensively upon the most pivotal findings, aligning with intuition and reason, which offer potential for generating robust managerial implications. Our focus extends beyond the principal impacts of individual parameters on tariff structure performance. We have identified compelling combinations that shed light on scenarios wherein three-part tariffs or menus thereof could outperform alternatives in terms of profitability, user participation, and usage quantity.

In light of the significant changes outlined above, our aim has been to demonstrate that the outcomes of our analysis are not trivial model artifacts, instead representing authentic insights. We believe that these revisions have substantially enhanced the quality of our research. We would like to convey our sincere appreciation for your patience and understanding throughout this process. Moreover, we are open to any additional comments or suggestions you may wish to provide.

For your convenience, we have enclosed the revised sections of our manuscript, specifically Section 5.3 and 5.4, which are also included in this response. Please refer to the subsequent paragraphs:

5.3 ***Comparison of different pricing structures***

*Better pricing strategies have been demonstrated to effectively segment the market. Offering a broader range of tariffs to consumers may also aid the service provider in catering to diverse needs and preferences, ultimately leading to higher customer satisfaction and loyalty, and potentially augmented revenue and profits. However, it’s important to acknowledge that providing a greater number of tariffs might also incur higher administrative or marketing costs.*

*Within this section, we undertake an analysis and evaluation of various tariff structures. Alongside examining the profitability of different tariff designs in the presence of overconfident consumers, our analysis also seeks to investigate the influence of implementing diverse pricing structures on total user participation and overall usage quantities.*

*In particular, we scrutinize the performance of the two-part tariff and the menu of three-part tariffs (including single, double, and triple optional three-part tariffs) within simulated experimental scenarios. Furthermore, several parameters, related to consumer behavior and market structure, are manipulated in the experiment. Factors that could potentially impact the efficacy of tariff structures, such as consumer preferences, price sensitivity, and the degree of overconfidence, are duly considered in this study.*

*To encapsulate consumer diversity, a sample of 1000 consumers exhibiting varying values of θ and λ has been employed. The parameter θ, symbolizing consumer preference, is assumed to follow a triangular distribution—a flexible distribution that is relatively easy to specify, with its support ranging between zero and one. It allows for both high and low means and variances in our experiment. Additionally, to explore the influence of price sensitivity heterogeneity on tariff structure profitability, the simulation study adopts a distribution of λ characterized by a triangular distribution with small and large variances. Although this setting might lead to demand functions for distinct consumers violating the Spence-Mirrlees single-crossing property, it better mirrors real-world situations where consumer behavior often displays significant heterogeneity. Lastly, triangular distributions are also employed to model the extent of overconfidence (with low and high means, and low and high variances). This strategy is embraced to more accurately capture the diversity in consumers’ levels of overconfidence and provide a more nuanced representation of real-world market phenomena.*

*Finally, to bolster the robustness of analysis, each of the 25 experimental settings (i.e., combinations of different parameters) is subjected to five replications in the simulation. This culminates in 160 observations for each of the four tariff structures. The details of the experimental design are delineated in Table 5, while Tables 6, 7, and 8 furnish the distributions of consumer preferences, overconfidence levels, and price sensitivity (i.e., variability of lambda) employed in the simulation.*

*Table 5. Experimental parameter setting*

| *Factors* | *Symbol* | *Number* | *Factor Levels* |
| --- | --- | --- | --- |
| *Mean of preference parameter* | *Mean* | *2* | *Low - High* |
| *Preference parameter variation* | *Variance* | *2* | *Large - Small* |
| *Mean of overconfidence parameter* | *OC Mean* | *2* | *Low - High* |
| *Variance of overconfidence parameter* | *OC Variance* | *2* | *Large - Small* |
| *Consumer price sensitivity* | *Lambda Var* | *2* | *Large - Small* |
| *Total number of environmental settings* | *2^5^ = 32* |  |  |
| *Number of replications* | *5* |  |  |
| *Total number of observations for each tariff structure* | *160* |  |  |
| *Total number of tariff structures* | *4* |  |  |
| *Total number of observations* | *640* |  |  |

*Table 6. Distribution of consumer’s preferences*

| *Mean* | *Variance* | *Preferences Distribution* |
| --- | --- | --- |
| *High* | *Large* | *Δ ( 0.4 , 0.7 , 1 )* |
| *High* | *Small* | *Δ ( 0.55 , 0.7 , 0.85 )* |
| *Low* | *Large* | *Δ ( 0.2 , 0.5 , 0.8 )* |
| *Low* | *Small* | *Δ ( 0.35 , 0.5 , 0.65 )* |

*Table 7. Distribution of consumer’s overconfidence level*

| *Mean* | *Variance* | *OC Distribution* |
| --- | --- | --- |
| *High* | *Large* | *Δ (0.1, 0.3, 0.5)* |
| *High* | *Small* | *Δ (0.25, 0.3, 0.35)* |
| *Low* | *Large* | *Δ (0.1, 0.2, 0.3)* |
| *Low* | *Small* | *Δ (0.15, 0.2, 0.25)* |

*Table 8. Distribution of consumer’s price sensitivity*

| *Lambda variance* | *Lambda* |
| --- | --- |
|  | *Distribution* |
| *Small* | *Δ ( 0.75 , 1.0 , 1.25)* |
| *Large* | *Δ ( 0.5 , 1.0 , 1.5)* |

5.4 ***Results of the experiment***

*To obtain the optimal pricing strategy across all tariff structures, we establish and conduct corresponding experiments entailing the simulation of various consumer distributions. The optimal pricing schemes, yielding the highest total expected profit, are determined by solving the mathematical programming problem using the genetic algorithm for each experimental setup and pricing structure. Additionally, the number of service subscribers and the total service consumption quantity linked to the pricing scheme leading to the highest expected profit are documented for subsequent analysis.*

5.4.1 *Analysis of profitability*

*To decompose the effects attributed to each individual factor on various performance measures (i.e., profitability, # of participants, and usage quantity), we employ analysis of variance (ANOVA) models. The ANOVA model outcomes are presented in Table 9. When using maximum profit obtained under different combination of experiment settings as the response variable, the results reveal significant impacts of pricing structures and experimental factors (i.e., mean and variance of overconfidence level, mean and variance of consumers’ preferences, and price sensitivity) on the profit of the service provider.*

*Table 9. Results of analysis of variance*

| *Main and interaction factors* | *Variance explained (%)* | | |
| --- | --- | --- | --- |
|  | *Profit* | *# of*  *participants* | *Usage*  *quantity* |
| *Mean* | *73.93*** | *1.86*** | *45.53*** |
| *Lambda_Var* | *2.98*** | *38.61*** | *4.16*** |
| *OC_Mean* | *15.08*** | *1.36*** | *3.64*** |
| *Pricing_Structure* | *1.63*** | *3.50*** | *2.22*** |
| *Variance* | *1.48*** | *12.89*** | *3.99*** |
| *OC_Variance* | *0.14*** | *0.59*** | *0.62*** |
| *Mean × Lambda_Var* | *0.35*** | *0.07* | *0.20*** |
| *Lambda_Var × OC_Mean* | *0.08*** | *0.04* | *0.02* |
| *Lambda_Var × Pricing_Structure* | *0.05*** | *0.62*** | *0.44*** |
| *Lambda_Var × Variance* | *0.11*** | *0.87*** | *0.69*** |
| *Lambda_Var × OC_Variance* | *0.01* | *0.01* | *0.00* |
| *Mean × OC_Mean* | *1.37*** | *0.01* | *0.06* |
| *Mean × Pricing_Structure* | *0.12*** | *1.05*** | *11.62*** |
| *Mean × Variance* | *0.28*** | *0.46*** | *0.55*** |
| *Mean × OC_Variance* | *0.10*** | *0.27** | *0.38*** |
| *OC_Mean× Pricing_Structure* | *0.31*** | *1.27*** | *7.02*** |
| *OC_Mean× Variance* | *0.04*** | *0.23** | *0.03* |
| *OC_Mean × OC_Variance* | *0.12*** | *0.55*** | *0.00* |
| *Pricing_Structure × Variance* | *0.23*** | *0.39* | *1.71*** |
| *Pricing_Structure × OC_Variance* | *0.05*** | *0.53** | *0.08* |
| *Variance × OC_Variance* | *0.06*** | *0.23** | *0.00* |
| *Note: * p < 0.05* | | | |
| *** p < 0.01* | | | |

*Specifically, the outcomes in Table 9 indicate that profit is likely influenced concurrently by mean preference, variance of preference, and level of overconfidence. Notably, the major driver influencing profits is the mean of consumers’ preferences (i.e., whether or not consumers possess a high mean preference for a service), which accounts for approximately 74% of variance in profit . This finding is consistent with previous studies that did not incorporate the overconfidence parameter (see, for example, Lin et al. [38]). Ultimately, market demand serves as a crucial profit determinant.*

*In the present study, introducing consumers’ overconfidence as an experimental factor also underscores the importance of consumers’ mean overconfidence level in influencing profitability, explaining around 15% of variability in profit. Given that this finding on the primary effect of consumers’ overconfidence aligns with our expectations, a service provider can enhance profits (or producer surplus) by targeting customers with high overconfidence levels.*

*Figure 3 illustrates that as consumers’ overconfidence levels rise, profits also increase, and vice versa. In our simulation experiment, service provider’s profitability experiences an approximate 40% boost when consumers’ overconfidence levels are high. This finding aligns with prior research such as Grubb [39] and Li et al. [12], which demonstrate an improvement in the service provider’s profit when consumer overconfidence is high.*

*While it’s intuitive that the degree of overconfidence in consumer preferences or behavior directly impacts service provider profits, the influence of overconfidence in tariff probability or performance is moderated by other conditions or factors. The true intrigue lies in how these boundary conditions affect tariff performance and yield valuable managerial implications.*

*`*

***Fig 3. Effect of overconfidence level on the profitability***

*It is crucial to emphasize that assessing solely the main effects of consumers’ preferences and overconfidence is insufficient when interaction effects are also at play, especially in cases where interactions occur between tariff structures and market environmental factors. Specifically, the mean preferences of consumers and their overconfidence levels play significant roles in the service providers’ profits for various pricing structures, as depicted in Figure 4.*

*In particular, when the mean preference is low and the overconfidence level is also low, a simpler tariff structure involving two price components should suffice. Consumers’ low preference and a lack of significant overestimation of their demand suggest relatively homogeneous demand among most consumers. This implies that there is no need for overly complex tariff structures such as a menu of three-part tariffs. This finding aligns with Bhargava and Gangwar (2018), who demonstrated that a three-part tariff can be simplified into a two-part tariff with zero allowance under certain market demand conditions.*

*However, it is worth noting that certain studies, such as Jang and Kwon [28] and Grubb [1], have highlighted the advantages of three-part tariffs in cases where consumers exhibit overconfidence or behavioral biases. In our study, we discovered that the menu of three-part tariffs can yield greater profitability than other pricing structures in specific combinations. Particularly, when consumers have low preferences but high overconfidence, pricing structures other than the two-part tariff appear to be superior choices. In this scenario, adopting an alternative tariff involving a menu of three-part tariffs could increase profits by approximately 15%, as depicted in Figure 4(a).*

*Nevertheless, if consumers' overconfidence levels are high, then regardless of whether their preferences are high or low, adopting a two-part tariff structure is no longer a reasonable option, and the profit disparities between the two-part tariff and other pricing structures become significantly distinct, as shown in Figure 4(b).*

***Fig 4. Interaction between consumer’s overconfidence level and pricing structure on the expected profit when the mean of preference is (a) low; or (b) high.***

*We can delve further into examining combinations of factors to extract useful managerial implications. For instance, as depicted in Figure 5, when the mean preference is high and the level of overconfidence is also high, although a more intricate tariff structure might appear suitable in theory, other considerations still warrant attention.*

*Indeed, in this scenario, if consumer preferences exhibit minimal variability (i.e., low variance of preference), as shown on the right-hand side of Figure 5(a), a two-part tariff structure could still prove effective since the overestimated demand might roughly satisfy the non-decreasing price elasticity (NPE) condition, a condition discussed in Bhargava and Gangwar* [40]*.*

*However, if the variance of preference is substantially high, a more intricate tariff structure would be necessary to accommodate diverse consumer needs. Specifically, when both the mean and variance of preference are high, the nonparallel lines in Figure 5(b) illustrate that with high overconfidence levels, introducing multiple tariff structures can achieve improved price discrimination and significantly influence a service provider’s profit potential. The larger gap between the two lines in the figure for a higher variance of preference emphasizes this effect. In such cases, two-part tariffs result in lower profits. Tariff structures involving three-part tariffs (whether one, two, or three optional three-part tariffs) tend to yield relatively higher profits. The service provider’s profitability decreases by around 13% when using a tariff with just two price components. This phenomenon becomes more pronounced when the variance of lambda (representing heterogeneity in price sensitivity) is also high.*

***Fig 5. Interaction between mean of preference and pricing structure on the expected profit when overconfidence level is high and the variance of preference is (a) small; or (b) large.***

5.4.2 *Analysis of total number of participants*

*While the service provider’s primary objective is to maximize revenues, other crucial metrics such as the number of customers who subscribe to the service and the amount of service consumed also require consideration. The second column of Table 9 presents the results of the analysis of variance when considering parameters related to consumer demand, the level of overconfidence, and tariff structure as input factors, with the total number of participants as the response variable. It becomes evident that all the (main) factors significantly affect the number of consumers subscribing to or using the service.* *Specifically, the results in Table 9 indicate that the variance of price sensitivity (i.e., λ) and the variance of consumer preference (i.e., O) are likely to play important roles in influencing the number of user participation, but their effects differ across various tariff structures.*

*Theoretically, when consumers are very homogeneous, all tariff structures might achieve a similar level of performance in attracting user participation, as shown in Figure 6. This means that when the variance of price sensitivity is small and the variance of preference is small, all tariff structures could potentially result in a higher number of users subscribing. However, when the variance of price sensitivity is large (regardless of whether variance of preference is large or small), other tariff structures will outperform the two-part tariff. The similar situation might arise when the variance of price sensitivity is small but the variance of preference is large.*

***Fig 6. Interaction between variance of preference and pricing structure on the total number of user when the variance of price sensitivity (i.e., lambda) is (a) small; or (b) large.***

*Indeed, the price sensitivity of consumers, represented by λ, significantly affects the total number of participating users, particularly when interacting with the mean and variance of preference, as illustrated in Figure 7. Specifically, when the variation of λ is small (indicating limited diversity in price sensitivity among consumers), and the mean preference is low, coupled with a high variance of preference, a two-part tariff pricing scheme fails to attract a substantial number of users to participate, irrespective of whether the level of overconfidence is high or low, as shown on the left-hand side of Figure 7(b).*

***Fig 7. Interaction between variance of lambda and pricing structure on the total number of user when the mean preference is low and the variance of preference is (a) small; or (b) large.***

***Fig 8. Interaction between variance of lambda and pricing structure on the total number of user when the mean preference is high and the variance of preference is (a) small; or (b) large.***

*The results presented in Figure 8 demonstrate that under the similar situation (i.e., when the variance of lambda is small, the variance of preference is large) but the mean preference is high, the results show that the two-part tariff try attracting more users than the menu of three-part tariff. In this scenario, even though consumers essentially have a high preference for the service, their demands vary considerably. If the service provider sets the tariff too high, there will essentially be an insufficient number of consumers subscribing to the service, thereby impacting the profit. In other words, the service provider needs to make a trade-off. By lowering the tariff, they can attract more users but will need to sacrifice earnings from consumers with very high demand. Figure 8(b) shows that if consumers have similar price sensitivity (i.e., with a small variance of price sensitivity), it is possible to implement a two-part tariff with slightly lower rates to encourage a larger number of user participation. However, this strategy becomes less effective when the variance of price sensitivity (λ) is larger.*

*On the other hand, when the more flexible three-part tariff is used, an allowance component in the tariff structure can better encourage consumer participation and profit extraction even in situations with significant heterogeneity among consumers. Thus, regardless of whether price sensitivity is high or low (i.e., irrespective of the degree of heterogeneity in consumers’ demand), the number of participants remains more stable.*

5.4.3 *Analysis of usage quantity*

*Table 9 shows that the mean of consumer preferences has a significant impact on the total usage quantity. Additionally, our numerical analysis reveals that the total usage quantity of consumers can also be influenced by the interaction between mean preferences and the overconfidence level, as well as the optimal tariff structures employed by the service providers. Specifically, the fixed fee and usage price can play a role in determining the usage quantity of consumers.*

*For a better understanding of the interactions between the overconfidence level, consumer preferences, and tariff structures on usage quantity, we can refer to Figure 9. In particular, Figure 9(b) shows that when consumers are highly overconfident and have high preferences, a two-part tariff with a high fixed fee and a high usage price will be implemented. Although the associated high fees will lead to a lower number of participants, a service provider can earn a high margin or unit profit from consumers, thus leading to higher overall profit. This is because highly overconfident consumers may be more likely to overestimate their future usage quantity and therefore will be induced to participate by a two-part tariff with a high fixed fee and high usage price (even though their actual consumption is much lower than what they expected).*

*In contrast, a flexible three-part tariff can take a wider range of consumers into consideration. For example, an appropriate allowance associated with the fixed fee can induce consumers with lower demand to participate while charging for additional units based on a usage price can induce high demand consumers. When facing highly overconfident consumers, the service provider will increase the fixed fee and at the same time lower the allowance. The service provider might also use a similar or even higher usage fee for each additional unit consumed. Since this tariff design will attract more participants, the overall usage quantity will increase as well.*

***Figure 9. Interaction between mean (level) of overconfidence and pricing structure on the expected total usage quantity when the mean of preference is (a) low; or (b) high.***

*Furthermore, when consumers have low preferences (i.e., lower demand) and are less overconfident, a two-part tariff structure with a lower fixed fee and a higher usage price will be implemented. By reducing the fixed fee, this tariff design can lead to a higher number of participants and a higher usage quantity, but the overall profit that can be extracted from the consumers will decrease, as shown on the left-hand side of Figure 9(a). However, when consumers have lower preferences but are highly overconfident, a two-part tariff structure with a higher fixed fee (and a higher usage price) will be implemented, thus leading to a lower usage quantity. Just as we can observe on the right-hand side of Figure 9(a), the usage quantities for different tariff structures under this situation are quite similar.*

**Reference**

1.	Grubb MD. Overconfident consumers in the marketplace. J Econ Perspect. 2015;29: 9–36. doi:10.1257/jep.29.4.9

12.	Li Y, Shan M, Li MZF. Advance selling decisions with overconfident consumers. J Ind Manag Optim. 2016;12: 891–905. doi:10.3934/jimo.2016.12.891

21.	Grubb MD. Selling to overconfident consumers. Am Econ Rev. 2009;99: 1770–1807. doi:10.1257/aer.99.5.1770

28.	Jang J, Kwon Y. Growing use of three-part tariffs by MNOs: understanding incentives of MNOs. 25th European Regional Conference of the International Telecommunications Society (ITS): “Disruptive innovation in the ICT industries: challenges for European policy and business.” Brussels, Belgium: International Telecommunications Society (ITS), Calgary; 2014.

38.	Lin S-W, Merdikawati S, Wu S-F, Yeh RH. Optimization and analysis of three-part tariff pricing strategies. OR Spectr. 2023. doi:https://doi.org/10.1007/s00291-023-00730-2

40.	Bhargava HK, Gangwar M. On the optimality of three-part tariff plans: When does free allowance matter? Oper Res. 2018;66: 1517–1532. doi:10.1287/opre.2018.1745

**Comments #2**

Model: Section 2.1 needs to be fully rewritten. The explanation of the consumer’s type is correct but very confusing. First, the WTP equations 3 and 4 are really the consumer surplus not the willingness to pay. Second, I think the consumer indexes add confusion instead to clarify the analysis. Third, the figure 1 need to be more clearly explained and connected to the model and the intuition of the model. Fourth, the indifferent consumer \theta* could be higher than \theta_2 as defined by table 1 and smaller than 1; as a consequence, you can have a third case that I think is necessary to analyze. Respect to section 2.2, I will move many of the calculations to the appendix. Also, I think you need to change the positions of section 3 and 4.

**Response to Comments #2**

Thank you for your comments. We appreciate your feedback on Section 2.1. Your input has been duly considered, and we have revised the relevant portions of this section to offer a clearer and more succinct elucidation of the consumer’s type. In response to your comments, we have addressed each point individually as outlined below.

**Comments #2a**

First, the WTP equations 3 and 4 are really the consumer surplus not the willingness to pay.

**Response to Comments #2a**

Thank you for drawing attention to this important point. From our perspective, the utilization of the WTP (Willingness To Pay) terminology in equations 3 and 4 is appropriate, as it offers a more direct measure of the value a consumer places on a good or service. Specifically, the WTP function take the consumer’s preference parameter $(O+r)$ into consideration and models map the consumer’s consumption quantity of a good or service to the total benefit or his/her willingness to pay for it. On the contrary, the “net” consumer surplus is calculated as the disparity between the maximum WTP and the actual price paid for the product or service. This general definition of consumer surplus represents the additional value the consumer gains from the acquisition beyond their expenditure. In our study, the “gross” consumer surplus (GCS) is depicted by the shaded region in Figure 1, aligning perfectly with the WTP measure.

Fundamentally, either the term “GCS” or “WTP” can be employed in Section 2.1, since both serve as identical metrics to estimate the total value consumers derive from a good or service, irrespective of the actual price paid. “WTP” seems even more straightforward and concise. Therefore, we concur that using the “WTP” terminology in equations 3 and 4 is appropriate. However, we also acknowledge the reviewer’s viewpoint that some readers might find “gross consumer surplus” a more familiar and comfortable term. To address this, we have revised the paragraph and introduced the term when first mentioning “willingness to pay.” Below is the revised version of the opening paragraph in Section 2.1.

*To formulate the consumer purchase decision and profit maximization, we use the original consumer type. We assume that the consumers face a monopolistic service provider offering a three-part tariff scheme comprising a fixed fee* $(T)$*, unit price* ${(p}_{0})$*, and usage allowance* $(q_{0})$ *under a fixed fee. We first define a consumer’s willingness to pay (i.e., gross consumer surplus) when a consumer decide to subscribe a service and consume a specific quantity or units of service. Below, we present two distinct cases for determining willingness to pay …*

**Comments #2b**

Second, I think the consumer indexes add confusion instead to clarify the analysis.

**Response to Comments #2b**

Thank you for your feedback regarding the consumer indexes. We apologize for any confusion they may have caused. While we maintain our belief in the utility of these indices for enhancing the analysis, we have chosen to present them differently. Specifically, instead of showcasing them in a table, we have opted to discuss them within the text itself, in a manner that is more intuitive and easier to comprehend. We trust that this approach addresses your concerns and contributes to the overall clarity of the discussion. For further details, please refer to the revised Section 2.1 on pages 9 to 10.

*To formulate the consumer’s willingness to pay (i.e., gross consumer surplus), we initially assume that the probability density of the original consumer type* $(O)$ *adheres to a continuous uniform distribution spanning a range of 0–1. Under specific* $\lambda$*, consumers with a type or preference that aligns with this uniform distribution can be categorized into three groups, based on two indices (i.e., two cutoff points):* $O_{1}$ *and* $O_{2}$*. These indices are linked to distinct three-part tariffs (i.e., associated with specific sets of (*$T$*,* $p_{0}$*,* $q_{0}$*)).*

*Particularly, the value of* $O_{1}$ *equals* $q_{0}$*, representing consumers of this type who will consume* $q_{0}$ *units when both the fixed fee (*$T$*) and unit price (*$p_{0}$*) are set at 0. The value of* $O_{2}$ *equals* $q_{0}+ \frac{1}{\lambda}p_{0}$*, representing consumers of this type who will consume* $q_{0}$ *units when the fixed fee (*$T$*) remains 0, but the unit price (*$p_{0}$*) exceeds 0. This implies that if a consumer’s type surpasses* $O_{2}$*, they are inclined to consume additional units (beyond the allowance) by paying a unit price of* $p_{0}$*, albeit only if the fixed fee is waived. Consequently,* $O_{2}$*-type consumers exhibit greater demand compared to* $O_{1}$*-type consumers.*

*Under the three-part tariff pricing scheme, the monopolistic service provider deals with three endogenous variables. The amount of the fixed fee* $(T)$ *will determine whether or not the consumers will subscribe to the service. If the fixed fee* $(T)$ *exceed the gross surplus derived from the consumer consumption, the consumers will not choose to subscribe. In such instances, the service provider can adjust the fixed fee* $(T)$*, unit price* $(p_{0})$*, and usage allowance* $\left( q_{0} \right)$ *to both optimize their profits and cater to consumer demands or requirements.*

*Figure 1 illustrates the three consumers groups. ...*

**Comments #2c**

Third, the figure 1 need to be more clearly explained and connected to the model and the intuition of the model.

**Response to Comments #2c**

Thank you for your feedback. We recognize that the explanation of Figure 1 needs further clarity and better integration with the model’s underlying concepts. We have revised the figure and the figure’s description to offer a more coherent explanation of its relevance within the model. Please kindly refer to the enhanced description on pages 10 to 11:

Fig 1. Three group of consumers and its gross consumer surplus for consumers with different $O^{*}$s.

*Figure 1 illustrates the three consumer groups determined by the consumer indices* $O_{1}$ *and* $O_{2}$*. In Figure 1(b), the first consumer group is portrayed—these are consumers whose preferences (or types) fall within the interval (0,* $O_{1}$*). Since* $O_{1}$ *represents consumers who would consume* $q_{0}$ *units under the condition of both zero fixed fee (T) and zero unit price (p_0), those with preferences lower than* $O_{1}$ *consume less than the designated usage allowance (*$q_{0}$*). This occurs even when the unit price is zero and despite their option to consume the full usage allowance. Specifically, consumers within this group, when overconfident, still opt not to fully utilize the usage allowance defined by the tariff in their actual purchase behavior.*

*Figure 1(c) portrays the second consumer group, whose preferences span from (*$O_{1}$*,* $O_{2}$*), and who consume precisely the usage allowance upon subscribing. This group refrains from exceeding the usage allowance as the additional benefit or utility gained is less than the unit price.*

*Finally, Figure 1(d) illustrates the third consumer group, with preferences ranging from (*$O_{2}$*, 1), who surpass the usage allowance. The consumption of this group deliberately exceeds the usage allowance, as the additional consumer surplus derived from consuming one unit of service surpasses the unit price. Consequently, they continue consumption until the marginal benefit is equal to the unit price.*

*While Figure 1 yields valuable insights into consumer preferences and decisions, it’s important to recognize that the efficacy of different tariff structures can fluctuate based on market conditions and consumer preferences. Therefore, service providers should use these insights as an initial guide for devising tariff structures and determining optimal pricing strategies tailored to their particular markets. In Section 5, we offer a numerical analysis showcasing how service providers can explore optimal pricing structures under specific market conditions..*

**Comments #2d**

Fourth, the indifferent consumer \theta* could be higher than \theta_2 as defined by table 1 and smaller than 1; as a consequence, you can have a third case that I think is necessary to analyze.

**Response to Comments #2d**

We appreciate your observation regarding the possibility of an indifferent consumer \theta* being higher than \theta_2 and smaller than 1. Basically, from a theoretical perspective, it is unnecessary to consider the third case or where the fixed fee T is larger than $\mathrm{CS}\left( O_{2} \right)$, in other words, consumer \theta* being higher than \theta_2 and smaller than 1. This is because according to the definition of “net” consumer surplus, a consumer will only purchase an extra service unit if the “net” consumer surplus is non-negative. Therefore, if the fixed fee T exceeds the gross consumer surplus of a consumer with type $O_{2}$, i.e., T is larger than $\mathrm{CS}\left( O_{2} \right)$, the consumer will not consume any extra service unit. By excluding this case, we can focus on the scenarios where consumers are willing to consume the services, i.e., the cases when T is less than $\mathrm{CS}\left( O_{1} \right)$ and when T is between $\mathrm{CS}\left( O_{1} \right)$ and $\mathrm{CS}\left( O_{2} \right)$. That is crucial for understanding the optimal pricing of three-part tariff in realistic scenarios.

In order to addresses this issue, we have addressed this important point in Section 2.1 on pages 11 to 12:

*The gross consumer surplus (willingness to pay) can be used to formulate the model of whether or not the consumers will purchase a service and the amount of service units that will be consumed, depending on the three-part tariff offered. We let* $CS\left( O \right)$ *be the function of the gross consumer surplus, with the overconfidence preference represented by the shaded area in Figure 1. Specifically,* $CS\left( O_{1} \right)= \frac{1}{2}\lambda\left( O_{1}+r \right)^{2}$ *is related to Equation (3) of consumer surplus when the overconfident consumer (*$O+r$*) preference is less than the allowance* $q_{0}$*. In addition,* $CS\left( O_{2} \right)= \left( O_{2}+r \right)\lambda q-\frac{1}{2}\lambda\left( O_{1} \right)^{2}$ *is related to Equation (4) of consumer surplus when the overconfident consumer (*$O+r$*) preference is larger than the allowance* $q_{0}.$ *As it is known, whether or not the consumers will subscribe to the service depends on the fixed fee* $(T)$*. Henceforth,* $O^{*}$ *will represent the original consumer type, which is indifferent about subscribing or not subscribing to the service.*

*When* $T$ *is less than* $CS\left( O_{1} \right)$*, the* $O_{1}$ *consumers will subscribe to the service, and the original consumer type* $O^{*}$ *will be less than* $O_{1}.$ *We divide all the consumers subscribing to the service into three parts. First, the consumers within the interval* $\left( O^{*}, O_{1} \right)$*consume* $O$*, that is, the quantity when the unit price is equal to 0. Second, the consumers within the interval* ${(O}_{1}, O_{2})$ *consume the exact usage allowance* $q_{0}$*. Finally, the consumers within the interval* ${(O}_{2}, 1)$ *consume* $O-\frac{1}{\lambda}p_{0}$*. When* $T$ *is between* $CS\left( O_{1} \right)$ *and* $CS\left( O_{2} \right)$*, the* $O_{1}$ *consumers will not subscribe to the service, and the original consumer type* $O^{*}$*will be within the interval* ${(O}_{1}, O_{2})$*. Then, we divide the consumers into two parts. First, the consumers within the interval* ${(O}_{1}, O_{2})$ *consume the exact amount of allowance* $q_{0}$*. Second, the consumers within the interval* ${(O}_{2}, 1)$ *consume* $O-\frac{1}{\lambda}p_{0}$*. Theoretically, we do not need to consider the case in which* $T$ *is larger than* $CS\left( O_{2} \right)$*, because the consumers will not consume any service unit if the fixed fee* $T$ *is greater than the consumer surplus, in other words, the consumer will only purchase a service unit if the consumer surplus is non-negative. Therefore, we focus mainly on the scenarios where the service is feasible and consumers are willing to consume the service, i.e., the cases when T is less than* $CS\left( O_{1} \right)$ *and when T is between* $CS\left( O_{1} \right)$ *and* $CS\left( O_{2} \right)$*.*

**Comments #2e**

Respect to section 2.2, I will move many of the calculations to the appendix. Also, I think you need to change the positions of section 3 and 4.

**Response to Comments #2e**

Thank you very much! We appreciate your suggestion to relocate certain calculations from Section 2.2 to the appendix. We have duly incorporated this adjustment into the manuscript. Additionally, regarding the proposal to interchange the positions of Section 3 and Section 4, we gave due consideration to this advice. However, since reviewer #1 initially recommended transferring Section 4 to precede Section 3, we have ultimately retained the current arrangement. Please find the relevant modifications we implemented on pages 14 to 16 of the revised manuscript.

**Comments #3**

Algorithm: The explanation of the algorithm has improved a lot, but I think the authors should connect it better with the model. For instance, it is not clear to me is the chromosomes are the thetas in the model and how those evolve and is related with the distribution of the types in the population.

**Response to Comments #3**

We appreciate your feedback and acknowledge the potential for enhancing the coherence between the algorithm and the model. To provide further clarity, it’s important to understand that the chromosomes within the algorithm correspond to the tariff structures. For instance, in cases where only a single three-part tariff is considered, these chromosomes comprise the fixed fee, usage price, and allowance. These elements are initially generated randomly from a uniform distribution, ensuring a diverse initial population with a broad range of values for each component. The goal of the optimization problem is to determine the optimum values for these components across various tariff structures, thereby maximizing the service provider’s profit.

Conversely, the notation $(O)$ in the model symbolize consumer preferences (of the market), modeled through a symmetric triangular distribution to encapsulate the variability or heterogeneity in these preferences. It’s important to note that this parameter is not encompassed within the chromosome structure, as it does not undergo optimization via the genetic algorithm. Instead, it functions as a parameter within the mathematical programming model.

To enhance the clarity when discussing the algorithm, we have refined the manuscript to establish a more coherent link between the algorithm and the model. Specifically, we have supplemented the relevant paragraph in Section 5.1 on pages 23 to 25 of the revised manuscript with additional discourse pertaining to this matter.

*The natural selection process described by Holland [31] in biology served as the basis for the development of the genetic algorithm (GA), which is a metaheuristic technique renowned for its ability to handle large search areas. Implementing a real-valued GA in many systems would be straightforward [32,33]. A population is first generated randomly by this algorithm. A group of individual solutions can be employed to characterize the population (chromosomes). In our model, these chromosomes comprise the fixed fee, the usage price and the allowance (i.e., the three key components of the three-part tariff). They are randomly generated from a uniform distribution to ensure the initial population of individuals possesses diversity, with a broad range of values for each component. The algorithm aims to find the optimal combination of these tariff components that maximizes profit by iteratively evolving the chromosomes across multiple generations. This progression involves the process of selection, crossover, and mutation, wherein chromosomes evolve and give rise to new offspring. This mechanism emulates the principles of natural selection, where the most adaptable individuals are likelier to pass on their genetic material to the next generation.*

*Within the selection process, a small subset of solutions from the current population is chosen by the algorithm to act as parents, based on the parents' fitness value, to form the basis for a new generation based on the parents’ fitness value. A chromosome is randomly selected from the population, its probability determined by the fitness function. Genetic mechanisms such as crossover and mutation generate the new generation. Once the termination condition is met, the best chromosome from the latest generation is presented as the final solution [34].*

*The number of generations, population size, crossover rate, and mutation rate are the important parameters typically included in GA programs to define and control the four core genetic operators associated with the algorithm [35]. The GA produces an efficient, optimal, or a nearly optimal solution depending on the parameters. The four important GA parameters we employ in our study are summarized below.*

1. *Generation: This parameter determines the total number of cycles (generations) necessary for the algorithm to complete. This study employs a termination criterion based on a maximum of 1,000 generations. During each generation, the algorithm assesses the fitness of each chromosome (tariff structure) based on its profit generation. Chromosomes with higher fitness hold a greater likelihood of selection for reproduction. Here, the individual with the best fitness value across 1,000 generations is identified as the optimal solution.*
2. *Population size: 50 chromosomes are randomly selected as the population of this study. A population’s size reveals details about its structure. According to De Jong and Spears[36], the ideal population size should fall between 50 and 100.*
3. *Crossover rate: The crossover rate denotes the likelihood of applying crossover (or recombination) to a pair of selected parent chromosomes to generate offspring chromosomes. Within the chromosome, where parental genetic material exchange occurs, a random gene is selected for crossover implementation. Subsequently, a new offspring is produced via crossover, contingent upon the chosen crossover point and specific parent components. We employ a real-value crossover in this study to choose the point at random, which then transfers the necessary components from the two parents to produce an offspring. The offspring can be determined as follows:*

$$o_{i}=\alpha\cdot x_{i}+\left( 1-\alpha\right)\cdot y_{i},$$

*(35)*

*where* $\alpha$ *is a random number with a uniform distribution between 0 and 1, and* $x_{i}$ *and* $y_{i}$ *are the two chosen gene-pool chromosomes.*

4. *Mutation rate: The mutation rate refers to the probability that a gene within a chromosome will undergo a mutation during the evolution process. Mutation often occurs after the completion of the crossover. The operator generates new adaptive solutions and helps prevent local optima by applying the modifications arbitrarily to one or more “genes” to create new offspring. Similar to a crossover, real-value mutation [37] is a method for producing new offspring from parents. The following can be used to determine the mutation rate:*

$$z_{i}=x_{i}+sign\cdot r_{i}\cdot\delta.$$

*(36)*

$Sign\in\{+1, -1\}$ *is chosen with equal probability,* $r_{i}=\rho\left( {range}_{i} \right), \rho\in\left[ 0.1, 0.5 \right].$*The range ratio (*$\rho$*), which is also known as the variable range ratio, is related to the maximum step that a mutation is permitted to produce;* ${range}_{i}$ *is the range of variable* $x_{i}$*; and* $t$*he parameter* $\delta$ *indicates the extent to which offspring can be produced from the parents and is expressed as follows:*

$\delta=2^{-k.u}$*,*

*(37)*

*where* $k\in N^{+}$*, and* $u\in[0,1]$*, with uniform probability. The mutation precision level necessary to reach the optimum depends on parameter* $k$*. However, in practice, the value of k is determined by the expected value of the mutation steps, and the higher the value of*$k$*, the more precise the resulting mutation operator. The GA operators employed in this investigation are described in detail below. To update the solution, we set*$\rho$ *to 0.1, signifying a low mean mutation step. Moreover, we set* $k$ *to 10, which complements the approach and is known to have a significantly strong influence on performance.*

**Comments #4**

Parametrization: The recognition of the limitations of the numerical analysis is necessary, but I still think that a discussion on how sensitive the results to the parameters of the model are is in place.

**Response to Comments #4**

Thank you for your feedback and for bringing this to our attention. We apologize for not fully addressing your previous request in our response. In the revised manuscript, we have incorporated your feedback and introduced a paragraph to acknowledge how sensitivity of our numerical analysis. We also mention that we performed the analysis with different parameter values and ranges. We are pleased to report that our experiments have consistently produced similar conclusions, which enhances the reliability of our findings.

We would also like to direct your attention to the added paragraph in the revised manuscript, which discusses the sensitivity of our findings to the model's parameters. Please find the added paragraph on page 41 of the revised manuscript:

*We acknowledge that the results and findings of our numerical analysis could potentially be affected by the specific parameter settings chosen for the analysis. To address this concern, we conducted a sensitivity analysis in our simulation study, employing varied ranges for the parameter distributions. Specifically, we adjusted settings related to the parameters (i.e., distributions used in the simulation experiments) and introduced variations of around 25% from their original values. Our conclusion reveals that these adjustments did not yield significant deviations in the outcomes. This suggests the robustness of our findings against parameter changes, strengthening the reliability of our study and mitigating the likelihood of findings arising solely from the model’s construction or parameter settings.*
